# Chronic pain and fatigue in multiple osteochondroma and Ollier disease, a systematic review

**DOI:** 10.1186/s12891-026-10025-6

**Published:** 2026-05-26

**Authors:** Ariane Kwiet, Brede Dammann, Trine Bathen

**Affiliations:** Norwegian Centre for Rare Diseases, Unit Sunnaas, Sunnaas hospital Bjørnemyrveien 11, Bjørnemyr, 1453 Norway

**Keywords:** Multiple osteochondromas, Ollier disease, Fatigue, Chronic pain, Systematic review

## Abstract

**Introduction:**

Multiple osteochondromas (MO) and Ollier disease are characterized by multiple tumours in the skeletal bones. They lead to bone deformity, restricted joint motion, interference with skeletal growth and increased risk of malignant transformation. The aim of this systematic review is to increase knowledge on chronic pain and fatigue in these diagnoses and identify gaps and topics for future research.

**Materials and methods:**

A systematic literature search was conducted in various databases in 2022 and updated in 2023. All types of studies were included that reported data on chronic pain and/or fatigue/vitality in patients with either multiple osteochondromas or Ollier disease. Study quality was assessed with Joanna Briggs Institute JBI’s critical appraisal tools.

**Results:**

A total of 63 studies (55 on multiple osteochondromas, 7 on Ollier disease, 1 on both), with a total of 1252 participants was found. The majority were case reports. No RCT was found. Fifty-six of 63 studies reported data only on pain; seven reported data on both pain and fatigue/vitality. Out of 63 studies, 29 used standardized assessment methods for pain, and seven for fatigue. Prevalence for chronic pain (when reporting non-site-specific pain) was reported as ranging from 80% − 95% for adults and 54–64% for children with multiple osteochondromas. Studies reporting fatigue showed a higher prevalence of severe fatigue or lower vitality scores when compared to other patient groups or the normal population. Pain and fatigue impacted ADL, mood, sleep, social and physical activities. Different interference factors for pain and fatigue were reported. Treatment strategies included “wait-and-see”, surgery, physiotherapy, and medication.

**Conclusions:**

Chronic pain and fatigue appear to be an important complaint in patients with multiple osteochondromas impacting the patient’s life. There is limited evidence on this topic for patients with Ollier disease. More research, particularly with larger cohort studies, is needed to be able to identify causation and associated factors, define optimal treatment and better understand the impact on patients’ life and their coping strategies.

**Trial registration:**

PROSPERO CRD42022379329.

**Supplementary Information:**

The online version contains supplementary material available at 10.1186/s12891-026-10025-6.

## Introduction

Multiple osteochondromas, also called multiple hereditary exostoses, and Ollier, disease also called enchondromatosis, are both rare diseases characterized by benign tumours in the skeletal bones. Multiple osteochondromas are defined by the presence of at least two osteochondromas, i.e. bone tumours with a cartilage cap [1]. Ollier disease is characterized by the presence of multiple enchondromas, i.e. cartilaginous tumours. These typically arise in the metaphysis of the bones [2].

Both syndromes affect children and adults in various ways. Common complications are bone deformity, restricted joint motion and interference with skeletal growth. In both conditions, there is a risk that one of the bone tumours undergoes a malignant transformation. The risk is higher in Ollier disease (described up to 50%) [3] than in multiple osteochondromas (around 2–5%) [1]. Additionally, in Ollier disease there is a risk for developing other type of cancers such as brain tumours or leukaemia [3]. Multiple osteochondromas are inherited in an autosomal dominant way. In around 90% genetic testing shows a mutation in either the Exostosin glycosyltransferase 1 gene (EXT1) or the Exostosin glycosyltransferase 2 (EXT2 gene) [1]. In Ollier disease, somatic mutations in the Isocitrate dehydrogenase 1 gene (IDH1) are found in the enchondromas [4], or Parathyroid hormone receptor 1 (PTHR1) in a small subgroup [5].

There is no medical treatment for neither multiple osteochondromas nor Ollier disease. Surgical procedures are used when osteochondromas or enchondromas cause medical complications or turn into malignant tumours.

A large part of literature focuses on genetics or surgical interventions. In the past few years, however, some studies suggested that chronic pain may be a problem in these diseases. Chronic pain interferes with all aspects of human living, such as physical, psychological and social well-being [6]. Chronic pain is defined as pain that lasts longer than three months [7]. Children and adults with chronic pain have increased risks for depression and anxiety, school or work absences, social isolation and lower quality of life [6].

Pain is often accompanied with fatigue [8, 9]. Fatigue is described as a multidimensional and complex phenomenon without a clear consensus on the definition [10, 11] and is often divided in physiological fatigue or fatiguability and experienced fatigue [12]. Experienced fatigue, often called subjective or perceived fatigue can be defined as: “an overwhelming sense of tiredness, lack of energy and feeling of exhaustion; mental, physical or both” [13]. Experienced fatigue can have large impact on daily life participation and quality of life in both adults [14] and children with chronic health conditions [15–17].

The objective of our study is to systematically review the literature on the topic of chronic pain and experienced fatigue in children and adults with multiple osteochondroma or Ollier disease. The study has three main goals: (a) present existing research on the topic of chronic pain and fatigue in patients, (b) make clinicians aware of current knowledge on chronic pain and fatigue and (c) identify gaps and topics for future research.

To our knowledge, no systematic review has been conducted on this topic before.

### Review questions

1. What do we know about chronic pain and fatigue in patients with multiple osteochondromas or Ollier disease?
2. Are chronic pain and experienced fatigue common in these patient groups? What types of pain can be identified in these patients?
3. Can we identify risk factors for developing chronic pain and/or fatigue?
4. What methods and tools are used to assess pain and fatigue in patients with multiple osteochondromas or Ollier disease?
5. What kind of treatments are being used to manage chronic pain and fatigue?

## Methods

The study protocol was registered in the International Prospective Register of Systematic Reviews (PROSPERO CRD42022379329). The Preferred Reporting Items for Systematic reviews and Meta-Analyses (PRISMA) checklist was followed [18].

### Eligibility criteria

#### Inclusion criteria

- We included all types of studies examining children and/ or adults with either the diagnosis multiple osteochondromas or Ollier disease.
- Studies were eligible if they assessed chronic pain, defined as pain lasting at least 3 month and/ or fatigue.
- Pain could be localised or generalized and measured with study specific or standardized pain measures.
- Fatigue could be assessed using study specific or standardized fatigue measures.
- Studies reporting separate data from the vitality scale of the Short Form 36 Health Survey (SF-36) or the RAND 36-Item Health Survey (RAND-36) were also included as the vitality scale has been widely used and validated as a proxy measure for fatigue in clinical research [13, 19–21].

#### Exclusion criteria

- Studies where osteochondromas or enchondromas are part of another syndrome such as trichorhinophalangeal syndrome type 2, metachondromatosis or Trevor disease.
- Studies where patients have one solitary osteochondroma or enchondroma. Studies with both solitary and multiple osteochondroma/ enchondroma were included if the authors reported separate data. If this was not the case, we contacted the authors to try to achieve separate data.

### Search strategy

The electronic literature search was performed by an academic librarian and included the databases MEDLINE (Ovid), Embase (Ovid), APA PsycInfo (Ovid), AMED (Ovid), CINAHL (Ebsco), Scopus (Elsevier), Web of Science (Clarivate) including A&HCI, ESCI, CPCI-SSH, CPCI-S, SCI-EXPANDED and SSCI. The search in MEDLINE included medical subject headings (MeSH) and search terms (synonyms) in title, abstract, author keyword for multiple hereditary exostoses and pain or fatigue.

The databases were searched from inception to present and were conducted in Dec 2022, with updated searches on 23rd Nov 2023. There were no restrictions by language, year, publication type or study design. The complete search strategies from alle the databases are given in additional file 1.

Contrary to more common diseases, only a few studies on rare diseases exist and these are often observational studies or case reports. We therefore found it important to include all available studies on this topic. We searched grey literature as well; relevant sources were for example reports from patient organizations. To find additional studies, we checked the reference lists and performed citation searching for all included studies. For this purpose, we used Science Citation Index, Scopus and/ or Google Scholar. The WHO International Clinical Trials Registry Platform (ICTRP) and ClinicalTrials.gov was searched to identify ongoing studies.

Literature search results were uploaded to an Endnote database and duplicates were removed.

### Selection process

We used Covidence, a web-based collaboration software platform that streamlines the production of systematic reviews. Titles and abstracts were screened independently by two review authors (A.K. and T.B.). Inter-rater agreement at this stage was 78.0%, with a Cohen’s kappa of 0.40. No disagreements requiring involvement of a third reviewer occurred at this stage. Full-text articles were subsequently assessed independently by the same two authors. Inter-rater agreement increased to 83.7% at the full-text stage (Cohen’s kappa = 0.64). Disagreements at the full-text stage were resolved through discussion and consensus involving the third author (B.D.)

### Data collection

All three authors independently extracted article data using a predefined data extraction sheet. The final data chart was agreed upon by consensus. Following data was extracted:

- Study design
- First author
- Publication year
- Journal
- Country where the study was performed
- Duration in months
- End of study period
- Number of participants
- Disease (multiple osteochondromas or Ollier disease)
- Patient characteristics (age, gender)
- Information on genetic mutation
- Pain prevalence, type of pain, pain intensity, mean duration of chronic pain
- Fatigue prevalence, fatigue score, fatigue intensity, mean duration of fatigue
- Methods and tools to assess pain and fatigue
- Treatment strategies and treatment outcomes
- Future research targets

### Quality assessment

All three authors independently rated the quality of all included studies. We found one study in which one of the review authors /TB) was also an author, and TB was excluded in quality assessment and data extraction of this study. We used JBI’s critical appraisal tools [22] to assess methodological quality of all included studies, using the checklists according to the type of study [23]. These tools contain an eight-item questionnaire for case reports and cross-sectional studies and a ten-item questionnaire for case series and qualitative research.

We then used a modification of the approach described by Zhang et al. [24], to categorize the studies as having high, moderate or low methodological quality. Studies with at least seven out of eight or nine out of ten “yes” were considered to be of high methodologic quality. Studies with four to six “out of eight or five to eight out of ten “yes” were considered to be of moderate methodological quality and less than four out of eight or less than five out of ten “yes” to be of low methodological quality. We modified the approach by Zhang et al. by counting “not applicable” items together with “yes” ratings, to avoid penalizing studies for criteria that were not relevant to their design or context.

There were cases where we were not sure if the condition was met and marked this with “unsure” in the tables. These were counted as “no”.

### Summary of findings and synthesis of results

We found great heterogeneity in the included studies, and the results are therefore mainly presented and synthesized narratively.

## Results

### Search results

**Fig. 1:**
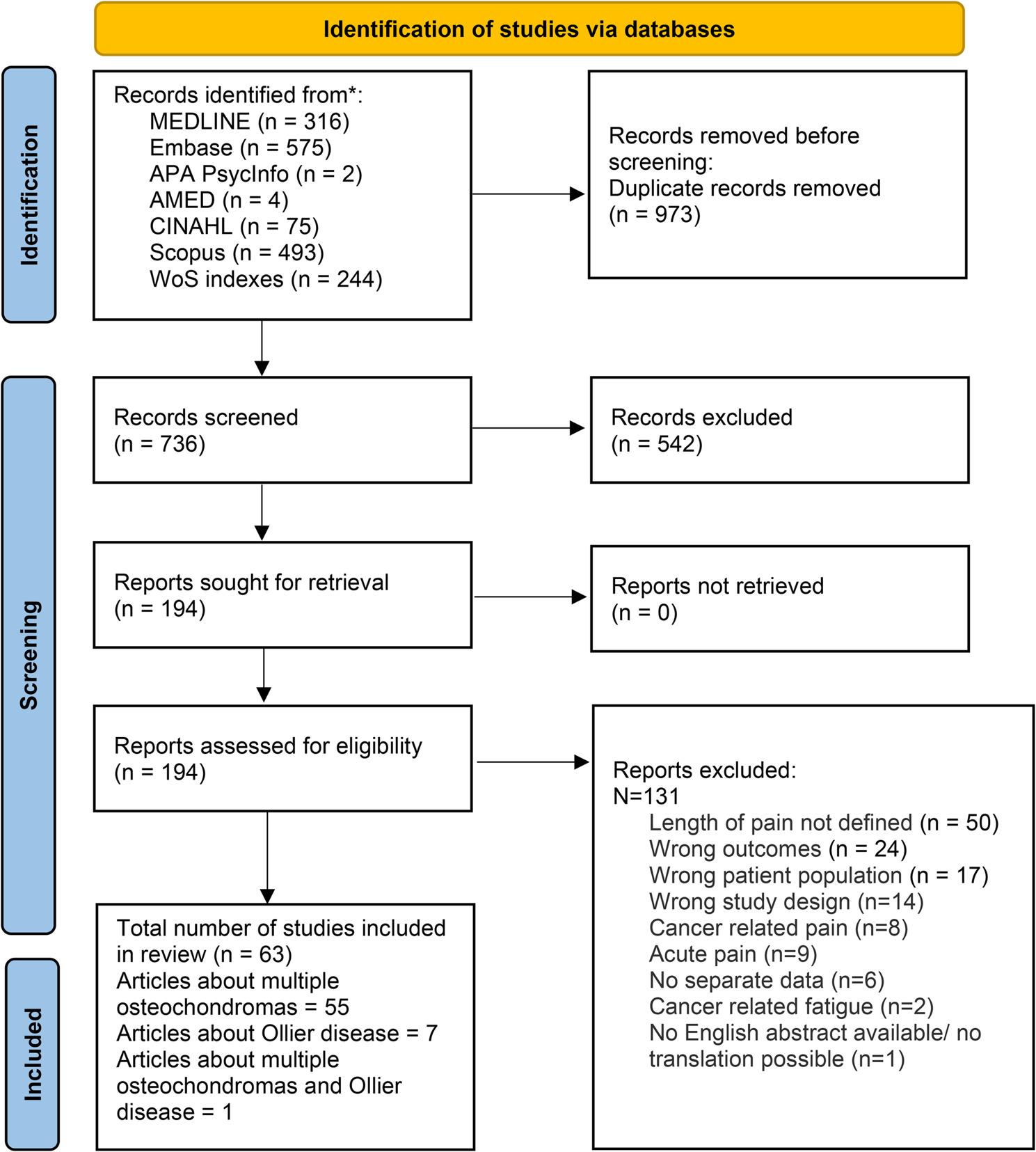
Study selection process. From: Page MJ, McKenzie JE, Bossuyt PM, Boutron I, Hoffmann TC, Mulrow CD, et al. The PRISMA 2020 statement: an updated guideline for reporting systematic reviews. BMJ 2021;372:n71. doi: 10.1136/bmj.n71

Figure 1 (PRISMA CHART) describes the study selection process that was carried out. Sixty-three studies were included in the study. None of the study authors who included patients with multiple osteochondromas or Ollier disease as part of a larger subgroup responded to the inquiry to provide separate results for these subgroups, therefore, all these studies had to be excluded.

Fifty-six of 63 studies reported data on pain, 7 reported data on both pain and fatigue/ vitality.

The retrieved studies were mainly in English, but we also found studies in French, Turkish, German and Spanish. One study in Chinese had to be excluded, as we found no way to translate it. Most studies included patients with multiple osteochondromas (see Fig. 1).

**Fig. 2:**
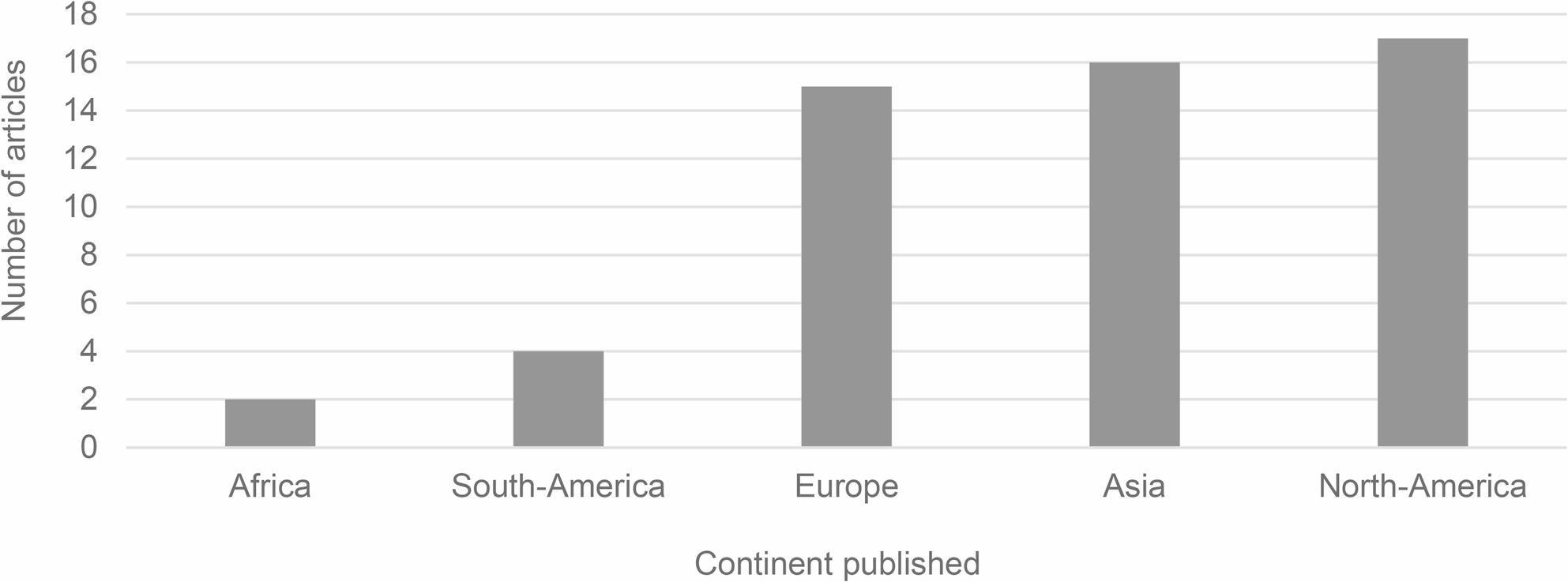
Number of articles published per continent

There was an almost equal distribution between Europe, Asia, and North America when looking at the continents from which the studies were published A few studies came from South America and Africa, but none from Australia (Fig. 2).

The studies were published between 1975 and 2023, we can see a considerable increase after 2014, Fig. 3.

**Fig. 3:**
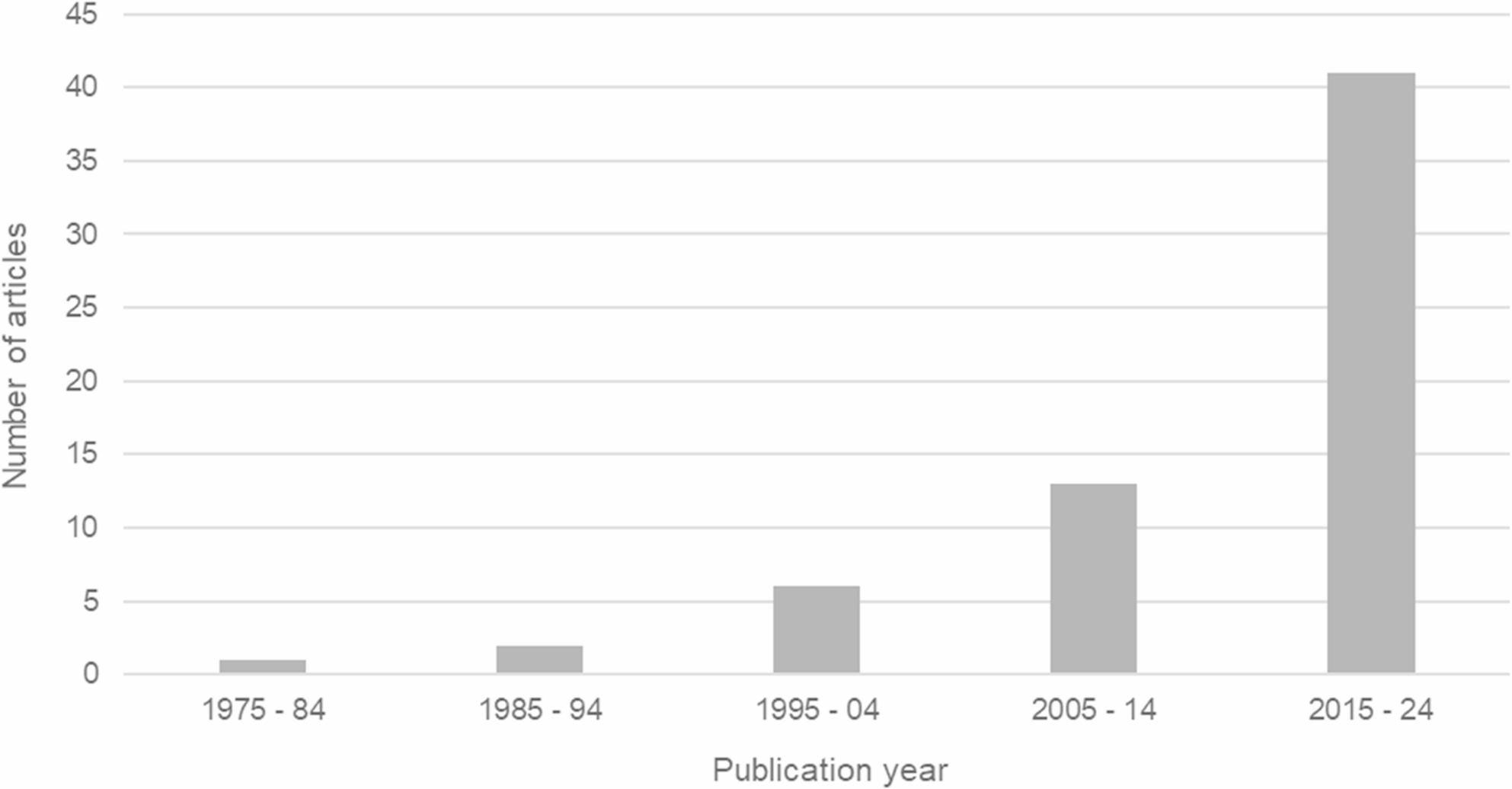
Number of articles on pain and fatigue published between 1975 - 2023

### Study design

**Fig. 4:**
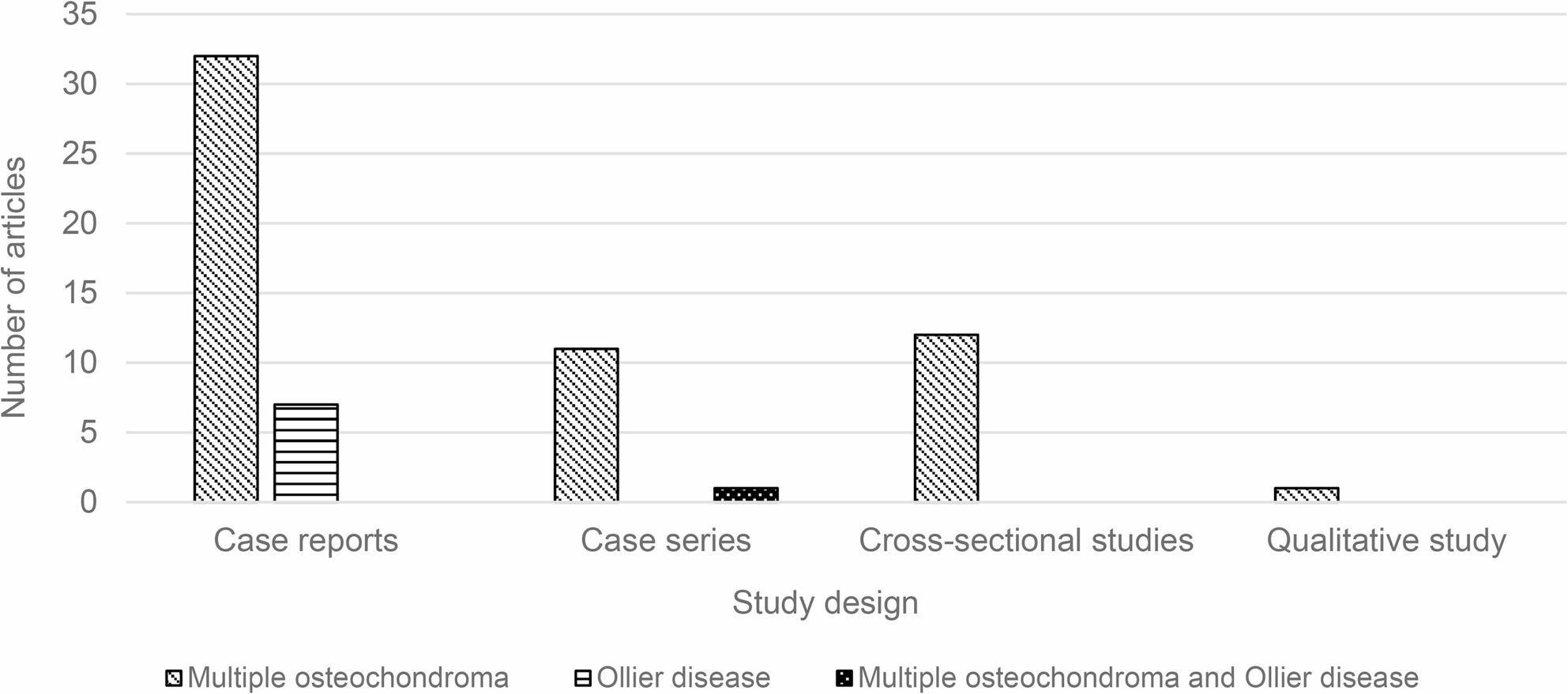
Number of articles in different study designs

Except for one study, all studies used quantitative methods as presented in Fig. 4. Most studies were case reports and case series. In addition, 12 cross-sectional studies were identified. No randomised controlled trials, cohort studies or systematic reviews were found. All studies on Ollier disease were case reports.

No other relevant reports were found that met the inclusion criteria. Only 32 out of 63 studies mentioned a study aim.

### Clinical information

In total, the included studies reported data on 1525 participants, the largest samples coming from the cross-sectional studies with patients with multiple osteochondromas. The distribution of participants in the different study types and diagnosis can be found in Table 1.

**Table 1:** Distribution of participants in different study types and diagnoses

Design type	Number of participants in total	Number of participants with MO	Number of participants with OD	Number of participants in mixed studies
Case reports	39	32	7	
Case series	84	82		2 (1of each)
Cross-sectional studies	1389	1389		
Qualitative research study	13	13		
Number participants in total	1525			

*MO*  Multiple osteochondromas, *OD* Ollier disease

The studies on patients with Ollier disease described 4 children and 3 adults. The studies with multiple osteochondromas patients described mostly adults (almost 60% of studies), followed by only children (almost 30%) and both children and adults (almost 14%). Some studies followed children into early adulthood for an evaluation of surgery methods. The study with both multiple osteochondromas and Ollier disease described two children.

Almost no study contained information about genetic mutations, which makes impossible to state anything about the correlation between specific mutations and prevalence or severity of pain or fatigue.

There was a large variation in the description of the clinical presentation of the patients. This was most evident in the case studies, where some authors mentioned pain only in one sentence and others provided a comprehensive description of the patient’s pain including severity, type and its interference with daily life.

### Assessment methods for pain and fatigue

We found no study who used a disease specific assessment tool for pain or fatigue.

#### Pain

Only 26 of 55 studies on patients with multiple osteochondromas used standardized assessment methods for pain (47%). Two of seven studies used standardized assessments of pain in patients with Ollier disease (29%), as well did the one study with both multiple osteochondromas and Ollier disease patients. Use of different assessment methods made comparisons difficult.

The most frequently used tool were the VAS or the NRS. Three studies used pain drawings [25–27]. Only Darilek et al. matched pain drawings with the existence of osteochondromas. Of those reporting pain, 55.1% reported generalized pain and 44.9% isolated pain which was localized to areas of osteochondromas [25]. In the cross-sectional studies, researchers often used multidimensional questionnaires, such as PedsQL paediatric pain questionnaire, SF-36, RAND 36 or Patient-Reported Outcomes Measurement Information System (PROMIS) bodily pain scale among others. Eleven studies used site-specific tools such as the Harris Hip score or the knee society score, but unfortunately, not all reported separate data for pain. Only one study (conference abstract) used an assessment tool for neuropathic pain (Douleur Neuropathique en 4 questions, DN4) and presents a large proportion of patients (47.6%) screening positive for neuropathic pain [2]. All used instruments can be found in Table 2.

**Table 2:** Standardized assessment methods for pain

Population	Assessment tool	Number of articles
Adults with multiple osteochondromas	RAND 36-Item Health Survey (RAND 36) bodily pain scale	1
	Short-Form Six-Dimension (SF-6D)	1
	36-Item Short Form Health Survey (SF-36)	2
	Presence of pain: yes/no	2
	Numeric rating scale (NRS)	5
	Pain drawing	3
	DN4 (questionnaire for neuropathic pain)	1
	Visual analogue scale (VAS)	7
	Disabilities of the Arm, Shoulder and Hand (DASH score)	1
	12-Item Short Form Survey (SF-12)	1
	Western Ontario McMaster Score, Knee Society Knee Score	1
	Harris Hip Score	4
	Knee Society Score	1
	Japanese Orthopaedic Association Hip score	1
Children with multiple osteochondromas	Children Health Questionnaire (CHQ PF-50)	1
	PedsQL paediatric pain questionnaire	1
	Numeric rating scale	1
	Pain drawing	3
	Faces Pain Scale-Revised	1
	Presence of pain: yes/no	2
	Visual analogue scale (VAS)	2
	Stanton and Hansen functional status scale Questionnaire	2
	12-Item Short Form Survey (SF-12)	1
	AOFAS ankle hindfoot scores	2
	36-Item Short Form Health Survey (SF-36)	1
	Paediatric Outcomes Data Collection Instrument (PODCI)	2
	Pain scale of 0 to 10	1
	Patient-Reported Outcome Measurement Information System (PROMIS): (Global Functioning and PROMIS domain scores for UE function, Pain, Depression, Anxiety, and Peer relations)	1
	Japanese Orthopaedic association Hip score	1
Adults with Ollier disease	Visual analogue scale (VAS)	1
	36-Item Short Form Health Survey (SF-36)	1
Children with Ollier disease	Visual analogue scale (VAS)	1
	Pain scale of 0 to 10	1

#### Fatigue

Seven studies used standardized assessment tools for assessing fatigue. All studies presenting results from standardized assessment on fatigue in multiple osteochondromas were cross-sectional studies [25–30]. There was one case study who used SF-36 vitality scale to present fatigue in one adult with Ollier disease [31]. One study examined both adults and children [27], while the other studies focused on fatigue in adults. The instruments used are described in Table 3.

**Table 3:** Standardized assessment methods for fatigue

Population	Assessment tool	Number of articles
Children with multiple osteochondromas	Paediatric Quality of Life Inventory Multidimensional Fatigue Scale (PedsQL MFS)	1
Adults with multiple osteochondromas	36-Item Short Form Health Survey (SF-36) vitality scale	2
	Short-Form Six-Dimension (SF-6D) vitality scale	1
	Fatigue Severity Scale	1
	Numeric rating scale (0–10)	1
	Checklist-Individual-Strength-Fatigue	1
	RAND 36-Item Health Survey (RAND 36) vitality scale	1
Children with Ollier disease	none	
Adults with Ollier disease	36-Item Short Form Health Survey (SF-36) vitality scale	1

### Narrative synthesis of reported results on pain and fatigue

#### Prevalence of pain

In 5 out of 9 cross-sectional studies reporting on no site-specific pain in patients with multiple osteochondromas, prevalence of pain was reported from 80% to 95% for adults [25–28, 30] and 54–64% for children [25–27, 30]. Because of the limited number of studies on Ollier disease, there is limited knowledge on prevalence of pain in Ollier disease.

#### Pain Interference

Twenty-six out of 55 studies on patients with multiple osteochondromas, 3 out of 7 studies on patients with Ollier disease and the one study on both multiple osteochondromas and Ollier disease described some kind of pain interference. Here, the authors described both interference with daily life activities such as school visits, work participation or participation in sports, as well as interference with ADL and physical activities. Some studies mentioned interference with mood and sleep. We found only one qualitative study, which described patients experiences with multiple osteochondromas [32]. Specific information is found in additional file 2 (data extraction).

#### Pain associated factors

Four cross-sectional studies described factors associated with increased pain. Three mentioned number of surgical procedures [25, 26, 33], as well as complications related to diagnosis [25] or deformities [26]. Other associated factors were age and problems at work in adults, negative perceptions of disease and problems at school in children [26], and membership in a support group [25]. See additional file 2 (data extraction) for separate data on each article.

#### Prevalence and severity of fatigue

Seven articles reported data on fatigue/vitality. Six of these reported data on a total of 679 patients with multiple osteochondromas [26–30, 34], while the seventh study was a case report on Ollier disease [31]. Only one study reported on fatigue in children [27].

Two studies using two different standardized fatigue measures in adults with multiple osteochondromas reported a high prevalence of severe fatigue, ranging from 55% [28] to 77% [27]. Both studies showed higher mean fatigue scores compared to those of the general population and patients with rheumatoid arthritis [27, 28]. One study also reported higher fatigue scores in 11 children with multiple osteochondromas compared to healthy children and children with short stature and rheumatoid arthritis [27].

Four studies reported data on SF 36/RAND 36 vitality scale in adults with multiple osteochondromas [26, 29, 30, 34], with mean values ranging from 52.0 to 65,5. Two studies found significantly lower vitality in multiple osteochondromas patients compared those of two general populations [26, 29].

One study using SF36 in a case report on rehabilitation in a patient with Ollier disease found large improvement on several SF 36 scales, including vitality [31].

#### Factors associated with fatigue

Only two studies investigated associations between fatigue and other variables. Amajjar et al. found a significant correlation between pain and fatigue [28]. Bathen et al. found higher fatigue in women, persons with higher age, high pain intensity and many pain locations (not significant) [27]. See additional file 2 (data extraction) for separate data on each article.

#### Treatment and treatment outcomes

The studies reporting on treatments and treatment outcomes vary considerably in in the level of detail provided. Six of seven case studies on Ollier disease described some kind of treatment. All of them focused on pain outcomes, only one reported fatigue outcome as well [31]. Four studies described different surgical interventions alone [35–37] or combined with physiotherapy [38], one described conservative treatment with analgesics [39] and one described a rehabilitation program [31]. Although all cases reported some benefit from the described therapies, only two studies used standardized tools for pain assessment [31, 38], the others only provided a description of pain and clinical examination.

One case series described two children, one with multiple osteochondromas and one with Ollier disease who were treated with bisphosphonates, and reported reduction in pain both on a pain scale and patient’s experience [40].

Forty-four of 55 studies on patients with multiple osteochondromas described a treatment, however, none reported fatigue outcomes. Three of the cross-sectional studies reported different treatment strategies that patients used, but without studying the effectiveness [25, 26, 41]. Nine case series assessed pain following surgical intervention, of which six used standardized assessment tools [34, 42–46], while the others described the clinical situation [47–49].

All case reports on patients with multiple osteochondromas described different treatment options, only 12 used standardized assessment of pain. Both conservative and invasive therapies were described. Surgical interventions ranged from corrective surgery, prosthesis or removal of osteochondromas. Other invasive methods included spinal cord stimulation or autologous fat grafting in respectively one case study or corticoid injections, often used prior to surgery. Conservative treatment included different kinds of pain medication, physiotherapy, observational (“wait and see”) strategies. In several case studies, particularly in adult patients undergoing prosthetic surgery, improvements were reported in both pain and functional outcomes. However, due to the heterogeneity of study designs, inconsistent reporting, and limited use of standardized outcome measures, it was not possible to systematically evaluate treatment effectiveness or compare clinical courses across studies. More detailed information about treatment and treatment outcomes can be found additional file 2 (data extraction).

#### Future research targets reported in included studies

Eighteen out of 63 studies mentioned targets for future research. The main message was that future studies with larger numbers of patients and longer follow-up were needed to provide more evidence about the prevalence, severity and interference of pain, as well as the best treatment options for pain in patients with multiple osteochondromas and Ollier disease. Little has been reported about fatigue.

### Quality assessment

As shown in Table 4 most of the studies were of moderate or high quality. Nevertheless, it is important to notice, that most of the studies were case series and case reports, meaning that they are ranged at the lowest end of the level of evidence [50]. We found some cross-sectional studies, but no studies of higher level of evidence were found.

**Table 4:** Quality assessment of included studies

Case reports	Low quality	Moderate quality	High quality
	3	17	19
Case series	0	5	6
Cross sectional studies	1	7	4
Qualitative study	0	1	0

When evaluating case studies, hardly any author mentioned the ethnicity of the patient, so we decided to give a “yes” in patients demographics, when age and sex was mentioned. Nevertheless, ethnicity is an important detail when interpreting case studies. A detailed description of quality assessment for each study can be found in additional file 3.

## Discussion

### Prevalence of pain and fatigue compared to the general population and other rare diagnoses

Chronic pain is described as one of the leading factors for disability and negatively influences patients in various ways [6]. Chronic pain is often associated with fatigue [9, 51]. This systematic review highlights the burden of chronic pain and fatigue for patients with multiple osteochondromas, both in childhood and in adulthood. For Ollier disease, the results are more unclear, with only 7 case studies found and one case series with one patient each. Although multiple osteochondromas and Ollier disease are distinct clinical entities, both are characterized by benign tumours in the skeletal system and are occasionally discussed together in the literature [52]. Given the rarity of these conditions and the limited available data—particularly for Ollier disease—we included both diagnoses to allow a broader exploration of shared clinical features such as chronic pain and fatigue. However, the marked imbalance in available evidence requires cautious interpretation, and conclusions regarding Ollier disease remain limited.

#### Pain

Prevalence for chronic non-site-specific pain was reported to be between 80% and 95% for adults with multiple osteochondroma [25–28, 30] and 54–64% for children [25–27, 30]. Additionally, a recently published study [53] reports a prevalence of 80% on chronic pain for adult patients with multiple osteochondromas which goes in line with the results from this systematic review. This is considerably higher than in the general population. Yong et al. described in his nationally representative study of adults in the USA, that 20,5% reported to suffer pain “most days or every day” [54]. Murray et al. conducted a systematic review and a meta-analysis in 2022 on the prevalence of chronic pain in young adults, in which 18.3% reported chronic localized musculoskeletal pain [55]. King et al. presented data on chronic pain in children and adolescents in a systematic review and showed that 4–40% of the children and adolescents suffered from musculoskeletal pain [56]. Higher prevalence of chronic pain is also described for other rare skeletal dysplasias such as achondroplasia [57] or osteogenesis imperfecta [58] or other rare diseases such as Marfan syndrome [59]. Sieberg et al. showed higher prevalence of chronic pain in several other rare diseases [60].

Regarding the type of pain, Darilek et al. described a high amount of generalized pain (55.1%) and raised the question if “there is an underlying mechanism for generalized pain” in multiple osteochondromas [25]. This is not answered by the studies that came after Darilek. Pain which is located at the site of the osteochondroma may be categorized as nociceptive pain (or neuropathic pain in case of nerve compression). It is possible that patients with multiple osteochondromas have a higher risk of developing nociplastic pain as well, hypothesized also by Amajjar et al. [53]. Murphy et al. described in their narrative review that nociplastic pain is often seen in patients with rheumatic diseases and uses bottom-up mechanisms as an explanation for these findings [61]. They describe bottom-up mechanism as “ascending pathways in the central nervous system, which are stimulated by peripheral inputs, leading to pain facilitation” [61]. A potential explanatory mechanism for the development of both localized and generalized chronic pain may be that repetitive irritation and pain because of the osteochondromas (and maybe enchondromas) may lead to sensitization and development of nociplastic pain. This could be researched further in future studies.

Amajjar et al. presented a high number of patients screening positive for neuropathic pain [28]. Out of our clinical experience, many patients with general pain, which would be classified as nociplastic pain, will present similar symptoms as patients with neuropathic pain and it is essential to combine a self-reported assessment tool with a clinical examination to distinguish between the different paint types. In their recent publication, Amajjar et al. are mentioning that “perhaps nociplastic pain and central sensitization play a more prominent role in MO” [53].

Unfortunately, only 7 case studies addressing chronic pain in patients with Ollier disease were identified. Therefore, it is not possible to state the prevalence of chronic pain in this population, which is considerably more rare than multiple osteochondromas. This may explain the limited number of studies found for our purpose. Due to the limited available data, it is not possible to draw firm conclusions regarding the prevalence or clinical significance of chronic pain in patients with Ollier disease.

#### Fatigue

Similar to the results on chronic pain, this study shows that patients with multiple osteochondromas suffer of more fatigue than the general population and other disease populations. The meta-analysis of Yoon et al. showed prevalence rate of 24.2% (95% CI, 19.9–29.5) for general fatigue in the global population [62], which is lower than reports of severe fatigue from 55% [27] to 77% [27] for patients with multiple osteochondromas in this review. Amajjar et al. published after the final literature search, report similar findings, providing contextual support for the observed high prevalence of fatigue [53]. There is a lack of studies on fatigue in children with multiple osteochondromas. Bathen et al. showed higher fatigue scores in 11 children with multiple osteochondromas compared to healthy children and children with short stature and rheumatoid arthritis [27], this should be verified in studies with a larger cohort.

There was only one case study addressing fatigue in a patient with Ollier disease [31]. This may reflect the rarity of the condition and the limited number of studies available. However, the available data are insufficient to draw conclusions regarding the prevalence or clinical significance of fatigue in this patient group.

Research on fatigue in general has been scarce across populations and conditions and has gotten less attention than research on pain [11]. Nevertheless, fatigue is proposed as one of the 10 most important research questions in other rare musculoskeletal conditions [63]. Therefore, further research on fatigue seems warranted also in multiple osteochondroma and Ollier disease.

### Use of standardized assessment tools

The use of standardized patient-reported outcome measures is recommended in rare diseases [64, 65]. In this review, many studies did not use standardized assessments tools. This makes it difficult to compare studies and treatment strategies. No study used diagnose-specific assessment tools, and, to our knowledge, there exists no assessment tools for either pain or fatigue specially designed for multiple osteochondromas or Ollier disease. Most assessment tools are either not disease-specific or are validated in more common diseases. Horn et al. have recently shown that it is possible to adjust and validate existing pain assessment tools (the Nordic Musculoskeletal Questionnaire) also in a rare disease [66].

#### Pain

The most commonly used assessment tool were the Visual Analogue Scale (VAS) and the Numeric Rating Scale (NRS) which have its limitation in evaluating chronic pain, as they do not capture the impact and consequences of the symptoms. However, both are quick and easy to use and can be very helpful before and after surgery or other treatments. Three studies used pain drawing, which is a useful tool to assess the location of the pain [25–27]. Only Darilek et al. matched a pain drawing with the existence of osteochondromas to further see at the relationship between the physical findings and the experienced pain [25].

All the cohort studies, and even some articles with other study designs used multidimensional questionnaires to assess pain. These questionnaires take longer time to complete than unidimensional assessment tools, but they are good methods to assess various pain-associated factors, such as mood disorders, pain variation, pain impact on daily live, among others [67].

#### Fatigue

A general problem in the research on fatigue in both common and rare disease is the lack of a clear definition for fatigue, the use of different assessment tools, that are designed to measure either fatigue or vitality, the use of different cut-off values for severe fatigue, the limited number of studies with fatigue as a main endpoint, and little focus on fatigue in children [10]. This is reflected in our study with few studies on fatigue and use of several different assessment tools, measuring either fatigue or vitality, making comparisons across studies difficult.

### Biopsychosocial model of pain and fatigue

Several studies described pain-related problems with daily life activities, sleep or mood as well as negative perception of disease as associated factors with pain. This is well known in other chronic pain conditions [6]. Fatigue was also reported to have strong correlation with pain and impact on quality of life. In a biopsychosocial model, both biological, psychological and social factors impact the development of chronic pain [68] and fatigue [69], and chronic pain and fatigue can impact these factors. A recent study published after the final literature search by Amajjar et al. [53] also reports a close relationship between fatigue and chronic pain, providing support for our findings. This suggest that both assessment and treatment of chronic pain and fatigue need to be multidimensional.

### Treatment strategies

Studies presenting treatment strategies varied considerably in their description. Surgery for a specific pain problem was often described in detail, but there is a lack of studies that describe conservative treatment strategies when surgery is not an option. Out of our clinical experience, patients born with their diagnosis and no perspective of getting rid of their diagnosis have other needs than patients with chronic pain diagnosis acquired later in life. We found no studies focusing on treatment strategies for fatigue.

In the qualitative study from Fraser et al., participants highlighted the lack of knowledge among both professionals and friends and family. They described their expectations from health care workers suggesting that professionals, among others, “should listen to the patient”, “take a holistic approach” and “take the condition seriously”. One patient mentioned that the health care workers should “not tell the patient that the condition is free from pain” [32]. As this study was conducted in 2000, patient experiences and expectations, as well as approaches to multidisciplinary pain management, may have evolved over time, and the findings should therefore be interpreted with some caution.

We have heard this statement also in our clinical experience. It is difficult for patients not to be believed and understood, causing a stress factor that can contribute to the development of chronic pain. Chou et al. have described similar needs in patients with chronic back pain [70].

Much of the knowledge on treating more common pain diagnosis can be applied to the management of patients with rare diseases. At the same time, it is important to acknowledge the specific features that rare diseases imply. Sieberg et al. present theories on how biopsychosocial aspects of rare diseases (stress because of delayed and incorrect diagnosis, little knowledge in health care workers, low self-esteem because of dysmorphism and more) can contribute and impact the development of chronic pain in children [60]. Being able to understand and accept fatigue has also been shown to be an important factor to enable patients to cope with fatigue [17, 71].

### Study design and quality

Most of the studies were of varying quality and belonged to the lowest level of evidence. Because of the heterogeneity of the studies, it was not possible to pool some of the results on pain or fatigue for statistical analysis. No study with the highest level of evidence was found.

Studies came mainly from Europe, Asia and North America, this may influence the results as cross-cultural differences in pain perceptions have been found [72].

### Implication for further research and clinical implications

These results may have implication both for further research and for clinical implications. Additional high-quality studies with larger cohorts are needed to gain more knowledge of the pathophysiology of chronic pain and fatigue in these populations, the pain types, associated factors and best treatment strategies. Qualitative research could contribute with insights on patient’s coping strategies.

When treating patients with multiple osteochondromas chronic pain and fatigue should be asked to evaluate the impact of these symptoms on the patients’ quality of life, physical and social activities and psychological health. In Ollier disease, the evidence is more unclear. However, asking the patient if pain and fatigue is a problem would be advisable.

### Strengths and limitations

As with other systematic reviews, this study is limited by the quality of the included studies and by incomplete reporting in several publications. The inclusion of all study types allowed a comprehensive overview over the published material. The predominance of case reports and case series introduces a risk of publication bias, as such designs are more likely to report unusual or severe presentations, particularly with respect to pain. This may lead to an overrepresentation of pain and inflated prevalence estimates. However, fatigue was more frequently assessed in cross-sectional studies, which may reduce the bias for this outcome.

Although the SF-36/RAND-36 vitality scale has been validated as a measure of fatigue, it was developed as part of a quality-of-life instrument and may represent a more general energy/fatigue construct, than fatigue-specific instrument. This may have influenced fatigue prevalence estimates. These factors should be considered when interpreting the findings.

The handling of “not applicable” items in the quality assessment, which were scored as fulfilled criteria, may introduce bias by potentially overestimating study quality.

The included studies were predominantly conducted in Europe, Asia, and North America, with limited representation from other regions. Cultural differences in pain perception and reporting may therefore affect the generalizability of the findings to underrepresented populations.

Inter-rater agreement statistics were not calculated for the quality appraisal stage, which may limit the formal assessment of consistency between reviewers, however, disagreements were resolved through discussion and consensus.

Strengths of this study was the detailed search strategy by a research librarian, the use of blinded study selection in Covidence by two independently working authors with a third author resolving conflicts, and blinded data extraction and quality assessment by three authors using a data extraction template and standardized quality appraisal tools.

## Conclusion

This systematic review highlights the burden of chronic pain and fatigue in patients with multiple osteochondromas. In Ollier disease, the evidence is less clear. There is still a large knowledge gap regarding the prevalence and impact of chronic pain and fatigue, the pathophysiology, pain types, causation and associated factors, and optimal treatment strategies. Health care workers treating these patients should consider these findings.

## Supplementary Information

Additional file 1.

Additional file 2.

Additional file 3.

## Data Availability

Data is provided within the manuscript or supplementary information files.

## Abbreviations

EXT1: Exostosin glycosyltransferase 1
EXT2: Exostosin glycosyltransferase 2
IDH1: Isocitrate dehydrogenase 1
PTHR1: Parathyroid hormone receptor 1
JBI’s critical appraisal tools: Joanna Briggs Institute critical appraisal tools
PROSPERO: International Prospective Register of Systematic Reviews
PRISMA: The Preferred Reporting Items for Systematic reviews and Meta–Analyses
SF: 36–Short Form 36 Health Survey
RAND 36: RAND 36–Item Health Survey
MO: multiple osteochondromas
OD: Ollier disease
PROMIS: Patient–Reported Outcomes Measurement Information System
DN4: Douleur Neuropathique en 4 questions
VAS: Visual Analogue Scale
NRS: Numeric Rating Scale
