# Supplementary material for: Chronic pain and fatigue in multiple osteochondroma and Ollier disease, a systematic review
Source: BMC Musculoskelet Disord. 2026 May 26;27:624. doi: 10.1186/s12891-026-10025-6 (PMC13393944; doi:10.1186/s12891-026-10025-6)
Supplement: Supplementary file 1 — Additional file 1. [file 12891_2026_10025_MOESM1_ESM.docx]

**Additional file 1**

**Documentation on the literature search for: Chronic pain and fatigue in patients with multiple osteochondroma and Ollier disease**

The following databases were searched:

| **Database** | ***Number of  retrieved records***  ***13.12.2022*** | **Number of retrieved records**  **23.11.2023** |
| --- | --- | --- |
| MEDLINE (Ovid) | *303* | 316 |
| Embase (Ovid) | *528* | 575 |
| APA PsycInfo (Ovid) | *2* | 2 |
| AMED (Ovid) | *3* | 4 |
| CINAHL (EBSCO) | *73* | 75 |
| Scopus (Elsevier) | *452* | 493 |
| A&HCI , ESCI , CPCI-SSH , CPCI-S , SCI-EXPANDED , SSCI (Web of Science, Clarivate) | *240* | 244 |
| Number of records before deduplication: | *1601* | 1709 |
| Number of duplicate records removed: | *925* | 973 |
| **Number of records after deduplication:** | *676* | **736** |

All searches were performed on 13 December 2022 by Academic Librarian Hilde Strømme, University of Oslo Library of Medicine and Science.

Updated search were performed on 23 november 2023 by Academic Librarian Hilde Iren Flaatten, University of Oslo. Library of Medicine and Science.

**Ovid MEDLINE(R) ALL**

1 Exostoses, Multiple Hereditary/
2 (familial exostos* or multiple exostos* or multiple hereditary exostos* or multiple cartilaginous
exostos* or multiple osteochondroma* or hereditary deforming chondrodysplasia* or diaphyseal aclasis or bessel-hagen disease or ext1 or "ext 1" or "exostosin-1" or ext2 or "ext 2" or "exostosin-2").tw,kf.
3 Enchondromatosis/
4 (enchondromatoses or (ollier* adj disease) or multiple enchondros* or multiple enchondroma* or hemangiomatosis chondrodystrophica or kast syndrome or dyschondroplasia or "chondrodysplasia with hemangioma" or chondroplasia angiomatosis or "enchondromatosis with hemangiomata" or "dyschondrodysplasia with hemangiomas" or "multiple angiomas and endochondromas" or multiple cartilaginous enchondromatos* or enchondromatosis).tw,kf.
5 exp pain/
6 pain*.tw,kf.
7 exp Fatigue/
8 (fatigue or lassitude or tiredness or vitality or exhaust*).tw,kf.
9 (or/1-4) and (or/5-8)

**Embase Classic+Embase**

1 hereditary multiple exostosis/
2 (familial exostos* or multiple exostos* or multiple hereditary exostos* or multiple cartilaginous exostos* or multiple osteochondroma* or hereditary deforming chondrodysplasia* or diaphyseal aclasis or bessel-hagen disease or ext1 or "ext 1" or "exostosin-1" or ext2 or "ext 2" or "exostosin-2").tw,kf.
3 Enchondromatosis/
4 (enchondromatoses or (ollier* adj disease) or multiple enchondros* or multiple enchondroma* or hemangiomatosis chondrodystrophica or kast syndrome or dyschondroplasia or "chondrodysplasia with hemangioma" or chondroplasia angiomatosis or "enchondromatosis with hemangiomata" or "dyschondrodysplasia with hemangiomas" or "multiple angiomas and endochondromas" or multiple cartilaginous enchondromatos* or enchondromatosis).tw,kf.
5 exp pain/
6 pain*.tw,kf.
7 Fatigue/
8 (fatigue or lassitude or tiredness or vitality or exhaust*).tw,kf.
9 (or/1-4) and (or/5-8)

**APA PsycInfo**

1 (familial exostos* or multiple exostos* or multiple hereditary exostos* or multiple cartilaginous exostos* or multiple osteochondroma* or hereditary deforming chondrodysplasia* or diaphyseal aclasis or bessel-hagen disease or ext1 or "ext 1" or "exostosin-1" or ext2 or "ext 2" or "exostosin-2").tw.
2 (enchondromatoses or (ollier* adj disease) or multiple enchondros* or multiple enchondroma* or hemangiomatosis chondrodystrophica or kast syndrome or dyschondroplasia or "chondrodysplasia with hemangioma" or chondroplasia angiomatosis or "enchondromatosis with hemangiomata" or "dyschondrodysplasia with hemangiomas" or "multiple angiomas and endochondromas" or multiple cartilaginous enchondromatos* or enchondromatosis).tw.
3 exp pain/
4 pain*.tw.
5 Fatigue/
6 (fatigue or lassitude or tiredness or vitality or exhaust*).tw.
7 (or/1-2) and (or/3-6)

**AMED (Allied and Complementary Medicine)**

1 (familial exostos* or multiple exostos* or multiple hereditary exostos* or multiple cartilaginous exostos* or multiple osteochondroma* or hereditary deforming chondrodysplasia* or diaphyseal aclasis or bessel-hagen disease or ext1 or "ext 1" or "exostosin-1" or ext2 or "ext 2" or "exostosin-2").ti,ab,et.
2 (enchondromatoses or (ollier* adj disease) or multiple enchondros* or multiple enchondroma* or hemangiomatosis chondrodystrophica or kast syndrome or dyschondroplasia or "chondrodysplasia with hemangioma" or chondroplasia angiomatosis or "enchondromatosis with hemangiomata" or "dyschondrodysplasia with hemangiomas" or "multiple angiomas and endochondromas" or multiple cartilaginous enchondromatos* or enchondromatosis).ti,ab,et.

3 exp pain/
4 pain*.ti,ab,et.
5 exp Fatigue/
6 (fatigue or lassitude or tiredness or vitality or exhaust*).ti,ab,et.
7 (or/1-2) and (or/3-6)

**CINAHL (EBSCO)**

S1 ("familial exostos*" OR "multiple exostos*" OR "multiple hereditary exostos*" OR "multiple cartilaginous exostos*" OR "multiple osteochondroma*" OR "hereditary deforming chondrodysplasia*" OR "diaphyseal aclasis" OR "bessel-hagen disease" OR ext1 OR "ext 1" OR "exostosin-1" OR ext2 OR "ext 2" OR "exostosin-2")
S2 MH "Enchondromatosis" OR (enchondromatoses OR (ollier* N0 disease) OR "multiple enchondros*" OR "multiple enchondroma*" OR "hemangiomatosis chondrodystrophica" OR "kast syndrome" OR dyschondroplasia OR "chondrodysplasia with hemangioma" OR "chondroplasia angiomatosis" OR "enchondromatosis with hemangiomata" OR "dyschondrodysplasia with hemangiomas" OR "multiple angiomas and endochondromas" OR "multiple cartilaginous enchondromatos*" OR enchondromatosis)
S3 MH "Pain+" OR pain* OR MH "Fatigue+" OR (fatigue OR lassitude OR tiredness OR vitality OR exhaust*)
S4 (S1 OR S2) AND S3

**Scopus (Elsevier)**

TITLE-ABS-KEY ((("familial exostos*" OR "multiple exostos*" OR "multiple hereditary exostos*" OR "multiple cartilaginous exostos*" OR "multiple osteochondroma*" OR "hereditary deforming chondrodysplasia*" OR "diaphyseal aclasis" OR "bessel-hagen disease" OR ext1 OR "ext 1" OR "exostosin-1" OR ext2 OR "ext 2" OR "exostosin-2") OR (enchondromatoses OR (ollier* W/0 disease) OR "multiple enchondros*" OR "multiple enchondroma*" OR "hemangiomatosis chondrodystrophica" OR "kast syndrome" OR dyschondroplasia OR "chondrodysplasia with hemangioma" OR "chondroplasia angiomatosis" OR "enchondromatosis with hemangiomata" OR "dyschondrodysplasia with hemangiomas" OR "multiple angiomas and endochondromas" OR "multiple cartilaginous enchondromatos*" OR enchondromatosis)) AND (pain* OR fatigue OR lassitude OR tiredness OR vitality OR exhaust*))

**Science Citation Index Expanded, Social Sciences Citation Index, Arts & Humanities Citation Index, Conference Proceedings Citation Index – Science, Conference Proceedings Citation Index – Social Science & Humanities, Emerging Sources Citation Index (Web of Science, Clarivate)**

TS=(((("familial exostos*" OR "multiple exostos*" OR "multiple hereditary exostos*" OR "multiple cartilaginous exostos*" OR "multiple osteochondroma*" OR "hereditary deforming chondrodysplasia*" OR "diaphyseal aclasis" OR "bessel-hagen disease" OR ext1 OR "ext 1" OR "exostosin-1" OR ext2 OR "ext 2" OR "exostosin-2") OR (enchondromatosis OR (ollier* NEAR/0 disease) OR "multiple enchondros*" OR "multiple enchondroma*" OR "hemangiomatosis chondrodystrophica" OR "kast syndrome" OR dyschondroplasia OR "chondrodysplasia with hemangioma" OR "chondroplasia angiomatosis" OR "enchondromatosis with hemangiomata" OR "dyschondrodysplasia with hemangiomas" OR "multiple angiomas and endochondromas" OR "multiple cartilaginous enchondromatos*" OR enchondromatosis)) AND (pain* OR fatigue OR lassitude OR tiredness OR vitality OR exhaust*)))
