# Supplementary material for: Chronic pain and fatigue in multiple osteochondroma and Ollier disease, a systematic review
Source: BMC Musculoskelet Disord. 2026 May 26;27:624. doi: 10.1186/s12891-026-10025-6 (PMC13393944; doi:10.1186/s12891-026-10025-6)
Supplement: Supplementary file 2 — Additional file 2. [file 12891_2026_10025_MOESM2_ESM.docx]

| Additional file 2. Data extraction of included studies on pain and fatigue in Ollier disease (OD) and multiple osteochondromas (MO) |
| --- |

| Study information: Title, lead author,  publications year | Study design | Study duration | Patient characteristics (age, gender), number | Assessment methods pain, both standardized and study specific | Assessment methods fatigue both standardized and study specific | Results for pain prevalence, type of pain, pain intensity, duration and pain interference | Results on fatigue prevalence, Mean fatigue score (SD) fatigue / vitality instruments, intensity duration | Treatment strategies and outcomes | Future research targets reported by authors |
| --- | --- | --- | --- | --- | --- | --- | --- | --- | --- |
| Ollier disease | | | | | | | | | |
| Multiple enchondromatosis: a case report  Benbouazza K 2002 | Case report | 3 years | 40 years, female  number: 1 | Description of pain and clinical picture |  | Generalized bone pain that was mechanical, moderate  10 years |  | The patient was given analgesics and non- nonsteroidal anti-inflammatory drugs, to some benefit.  At follow up after three years clinical and radiologic abnormalities were unchanged. |  |
| Enchondromatosis affecting the foot: A case report  Edwards SR 2021 | Case report | approximately 1 year | 16 years, male  number: 1 | Description of pain and clinical examination |  | Increasing pain and disfigurement of second and third toes 18 months preventing him from participating in sport |  | Surgery Six weeks postoperatively patient reported no pain, and toes were no longer deformed. This continued up until his discharge appointment at 12-months postoperatively. |  |
| Rehabilitation experience in a case of Olliers disease Formis A 2003 | Case report | One month | 32 years, male  number: 1 | Visual analogue scale for pain, SF 36 physical pain scale Description of pain history and clinical examination. | SF 36 vitality scale | headache, chronic low back pain increased after back extension  VAS 5  SF 36 physical pain scale 21%  general weakness with autonomy reduction in activities of daily living (ADL)  The patient was under high doses of analgesic drugs. | SF-36 vitality 25% | The patient took analgesic drugs and underwent physical therapy. Rehabilitation treatment lasted one month. Pain VAS 1.5, SF 36 physical pain 73%, fatigue (vitality SF-36) 90%, dosage of analgesic drugs was significantly reduced. | It is hopeful that in the future more rehabilitation studies on patients suffering from Ollier disease will be performed. |
| Multiple enchondromatosis: a case report and review of the literature Jacobs A 1975 | Case report and non-systematic literature review | approximately 1 year | 9 years, female  number: 1 | Description of pain and clinical examination |  | Painful swelling unable to tolerate footgear and walked with significant discomfort |  | Surgery  The patient was discharged in satisfactory condition, walking well. No report on post-surgery pain. |  |
| Three different methods for treating multiple enchondromatosis in one hand  Lu H 2015 | Case report | Operation and four-year follow-up | 15 years, male  number: 1 | Description of pain and clinical examination |  | Pain in the left hand half a year |  | Surgery Three days after the surgery, the patient was able to perform full motion of the operated hand. No report on post-surgery pain. |  |
| A rare case of enchondromatosis of the knees and hands with involvement of Hoffa's fat pad and peri-articular soft-tissues  Sutera R 2013 | Case report |  | 56 years, male  number: 1 | Description of pain and clinical examination |  | Knee pain associated with functional limitation and soft tissue swelling in the left knee and both hands several years |  |  |  |
| Local design and manufacturing of patient-specific implant using Anatomage Medical Design Studio software: proof of concept - Botswana's 1st case report  Nkhwa 2023 | case report | surgery and 18 month follow-up | 14 years, female  number: 1 | VAS  Description of pain and clinical picture |  | VAS: 8/10 low back pain |  | surgery with pasient specific 3D printed implant, 12 weeks fysiotherapy from day 4 after surgery  painfree, good physical function 18 month post op. |  |
| Multiple osteochondroma and Ollier disease | | | | | | | | | |
| Bisphosphonates for pain management in children with benign cartilage tumors Winston MJ 2012 | Case series | Intervention and one year follow up | Case 1: Ollier disease, 12 years, female Case 2: MO 12 years, male number: 2 | pain scale of 0 to 10 before and after treatment Description of pain and clinical examination, patient report of experiences. |  | Case 1: majority of pain in left shoulder and leg; Interference with school and need of pain medication Case 2: intermittent pain right upper arm, feet, ankles, and lower legs. Interference with walking long distances and running. Need for pain medication. |  | Bisphosphonates.  Case 1: After therapy, the child went 6 to 7 months before missing a day of school. 3/10 Case 2: dramatic decrease in the amount of over-the-counter analgesics used 2/10. Both patients reported self-experienced improvement in pain and daily interference, | Further research is required to see whether our positive experience can be projected to other paediatric disorders associated with bone lesions causing intractable pain. |

| **Multiple osteochondromas** | | | | | | | | | |
| --- | --- | --- | --- | --- | --- | --- | --- | --- | --- |
| Buttock Pain and Sciatica Caused by a Femoral Osteochondroma; Akinyemi OA 2017 | Case report | Three-month follow-up | 26 y, female number: 1 | Numeric Pain Rating Scale |  | Lower back and buttock pain  NPRS at first assessment 8/10 Duration: 10 months prior first contact Interfered with gait and sitting position |  | Surgery  Three months post-surgery reduction to 2/10 on the NPRS |  |
| Bilateral scapular osteochondroma in Multiple Hereditary Exostosis patient presented with bilateral shoulder pain treated with arthroscopic and open excision: Case report; Alshayan FA 2021 | Case report | Not reported | 24 y, male;  number: 1 | Patient report of pain and limitations in daily activities, investigation of painful range of motion |  | Pre surgery: painful crepitation for three years. Pain interfering with his daily activities including sleeping in supine position Duration: 3 years |  | Non-surgical management including analgesia and physical therapy  didn’t' improve patient's pain.  After surgery: patient exhibited full painless range of motion with no crepitation |  |
| **Study information: Title, lead author,  publications year** | **Study design** | **Study duration** | **Patient characteristics (age, gender), number** | **Assessment methods pain, both standardized and study specific** | **Assessment methods fatigue both standardized and study specific** | **Results for pain prevalence, type of pain, pain intensity, duration and pain interference** | **Results on fatigue prevalence, Mean fatigue score (SD) fatigue / vitality instruments, intensity duration** | **Treatment strategies and outcomes** | **Future research targets reported by authors** |
| The assessment of fatigue and pain in multiple osteochondromas: A Dutch Cohort Study; Amajjar I 2022 | Cross-sectional study. Conference abstract | Not reported | Mean age 41.86 y (± 16.28), gender distribution not reported number: 342 (of 386 invited) | Numeric rating scale (0-10) and DN4 (not explained) | "Numeric rating scale (0-10) and Checklist-Individual-Strength-Fatigue (CIS; range = 8â€“56)". The results were compared with reference scores of normative data of healthy subjects, and patients with Rheumatoid arthritis (RA) and Chronic Fatigue syndrome (CFS). | Almost 80% of the patients reported pain  47,6% neuropathic pain Mean NRS = 4.50 ± 3.19 | Fatigue reported by 90.8% Severe fatigue (CIS-score> 35) by 55.1%. Mean CIS-score= 36.21 ± 7.98 mean NRS = 5.46 ± 2.99 significant association between fatigue and pain.  Significant higher fatigue- scores in MO patients. |  | These results evoke further research and clinical attention aimed at reducing pain and fatigue in patients with MO |
| Fatigue and pain in children and adults with multiple osteochondromas in Norway, a cross-sectional study; Bathen T 2019 | Cross sectional study | Not reported | Children: Median age 10.7 y (range 6-16), 7 boys, 4 girls, number: 11 Adults: Median age 37.1 y (range 21-67 years), 8 men, 13 women, number: 21 | Children: PedsQL paediatric pain questionnaire Adults: Presence of musculoskeletal pain last week; yes/no, pain intensity last week on Numeric pain rating scale (NPRS), pain location by drawing | Children: PedsQL Multidimensional Fatigue Scale (PedsQL MFS) Adults: Fatigue Severity Scale | Children 6/11  Adults 20/21 reported pain  Children: Median worst pain. Intensity 4.0 (0 - 9.5). Adults: Median pain intensity 6.5 (2 - 9) Children: median number of pain locations was 5. Adults: median number of pain locations was 5.5 (3-10). | Children: prevalence not reported. Adults: 15/21 reported severe fatigue (FSS ≥5)  Children: mean PedsQL MFS total score 65.53 (SD 20.95), significantly higher compared with other groups  Adults: FSS mean score 5.6 (SD 1.12), significantly higher compared with other groups |  | There is a need for further research on prevalence, possible causes and treatment for both fatigue and pain in children and adults with MO. Qualitative studies exploring the experience of living with pain and fatigue in persons with MO are needed. |
| Effect of Distal Ulna Osteochondroma Excision and Distal Ulnar Tether Release on Forearm Deformity in Preadolescent Patients With Multiple Hereditary Exostosis; Belyea C 2020 | case series |  | Mean age at time of surgery 7,9 years (Range 4.4-10 y), mean age at follow-up 16,1 y. 4 girls, 2 boys. number: 6 patients (7 forearms) | Visual analogue scale (data reported as follow-up results) Prescence of pain yes/no |  | On initial presentation, 66% of patients reported pain as their chief complaint. |  | Surgery Fifty percent of the cohort reported no pain in the operative upper extremity at final follow-up. At final follow-up, mean VAS pain score was 1.00 (1.16) |  |
| **Study information: Title, lead author,  publications year** | **Study design** | **Study duration** | **Patient characteristics (age, gender), number** | **Assessment methods pain, both standardized and study specific** | **Assessment methods fatigue both standardized and study specific** | **Results for pain prevalence, type of pain, pain intensity, duration and pain interference** | **Results on fatigue prevalence, Mean fatigue score (SD) fatigue / vitality instruments, intensity duration** | **Treatment strategies and outcomes** | **Future research targets reported by authors** |
| Preadolescent Patients With Multiple Hereditary Exostosis Benameur H 2022 | Case report | 5 months | 29 y, female number: 1 | Visual Analogue Scale (VAS), Disabilities of the Arm, Shoulder and Hand (DASH) score (2 questions about pain), but no separate data given |  | VAS pain (intensity) 3 at presentation Duration: 7 months |  | Surgery The pain score was 2/10 at three months after surgery |  |
| Timing of forearm deformity correction in a child with multiple hereditary exostosis; Beutel BG 2014 | Case report | 2 years | 11 - 13 y, male number:1 | Description of pain and clinical examination |  | aching sensation throughout the elbow without radiation |  | Surgery At his first postoperative visit, 9 days after surgery, pain was well-controlled, and left elbow ROM was from 15° to 90° of flexion/extension. | Larger prospective studies are needed to confirm these findings in the broader population of patients with MHE. |
| Joint pain in hereditary multiple exostoses; Bruce DJ 2015 | Case report |  | 72 y, male number: 1 | Description of pain and clinical examination |  | At presentation: Pain and stiffness in right hip, gradually worsening for many years. |  | Hip replacement, physiotherapy Two months post operation ROM and mobility improved. No report on pain |  |
| Osteochondromatosis: clinical variability and factors related to quality of life in children and adults; Caino S 2022 | Cross sectional study |  | median age 13.4 y (range 2,2 - 55.4y), 39 males, 27 females,  number: 66, 47 children, 19 adults | Children: Faces Pain Scale-Revised, FPS-R. Adults: SF 36 bodily pain scale Study specific survey for pain the past month, including the following variables: presence or absence of pain, severity (Faces Pain Scale-Revised, FPS-R),22 site, characteristics (somatic or neuropathic pain), pain frequency, and treatment | Children none. Adults SF-36 vitality scale | Pain referred by 30/47 children and 17/19 adults. Most common pain type was somatic pain. Pain severity varied: 35% mild; 27.5%, moderate; 37.5%, severe. In adults SF36 mean bodily pain score: 48.82 (SD 25.46) Pain affected routine activities in 22/28 children and 10/16 adults. | In adults SF 36 mean vitality score: 62.74 (43.91) |  |  |
| **Study information: Title, lead author,  publications year** | **Study design** | **Study duration** | **Patient characteristics (age, gender), number** | **Assessment methods pain, both standardized and study specific** | **Assessment methods fatigue both standardized and study specific** | **Results for pain prevalence, type of pain, pain intensity, duration and pain interference** | **Results on fatigue prevalence, Mean fatigue score (SD) fatigue / vitality instruments, intensity duration** | **Treatment strategies and outcomes** | **Future research targets reported by authors** |
| Health-related quality of life in people with hereditary multiple exostoses; Chhina H 2012 | Cross sectional study | 3 years, 2 month | Children: mean age 9.93 y (±3.48 y), 23 male and 40 female. Adults: mean age: 40.10 y (±13.01y), 19 male and 28 female number: 100, 43 children, 57 adults | Children: Children Health Questionnaire (CHQ PF-50), parent report, including bodily pain scale. Adults: SF-6D and SF-36, including bodily pain scales | Children none. Adults SF-36 and SF_6D vitality scale | CHQ PF-50 for children lower than US normative data; particularly in bodily pain and emotional self-esteem. Adults: Mean SF-36 Bodily pain score: lower than US and Canadian normative data  Adults: Twenty-six percent pain interferences with work a little bit of the time. | Adults: Mean SF-36 vitality score: 52.0, lower than US and Canadian normative data (60.9 and 65.8 respectively). SF-6: 20 (35%) reported vitality to be having a lot of energy most of the time. |  |  |
| Subacromial impingement syndrome in hereditary multiple exostoses; Craig EV 1986 | Case report | unsure, follow up 15 months after surgery | 32 years, female number: 1 | Description of pain and clinical examination |  | progressive left shoulder pain Duration: approximately one year  increasing in severity, unremitting and interrupting sleep. |  | steroid injections, exercise, anti-inflammatories with no effect, surgery At 15 months follow-up patient was pain free. |  |
| Gradual ulnar lengthening in children with multiple exostoses and radial head dislocation: results at skeletal maturity; D'Ambrosi R 2016 | Case series | unclear | Average age at surgery 10.1 y (range 8-12y), average age at follow-up 18.2 y (range 17 - 23y), 8 male and 7 female number: 15 | Moreover, each patient was asked to rate the function of the extremity according to specific criteria as described by Stanton and the SF-12 to assess their own quality of life VAS not described in methods, but under result? |  | Preoperative VAS severity mean 8.2 (range 7-9)  Preoperative patient pain function score was 1.6 (range 0 - 2) SF 12 results on bodily pain not stated separately |  | Surgery  VAS scores improved significantly from an average preoperative of 8.2 (range 7 - 9) to 2.3 (range 1 - 4). Patient pain function score improved significantly after surgery. |  |
| **Study information: Title, lead author,  publications year** | **Study design** | **Study duration** | **Patient characteristics (age, gender), number** | **Assessment methods pain, both standardized and study specific** | **Assessment methods fatigue both standardized and study specific** | **Results for pain prevalence, type of pain, pain intensity, duration and pain interference** | **Results on fatigue prevalence, Mean fatigue score (SD) fatigue / vitality instruments, intensity duration** | **Treatment strategies and outcomes** | **Future research targets reported by authors** |
| The impact of hereditary multiple exostoses on quality of life, satisfaction, global health status, and pain; D'Ambrosi R 2017 | Cross-sectional study |  | Mean age 40 y (range 17-81y), 62% females, 38% males number: 50 | Visual Analogue Pain Score (VAS) Short-Form Health Survey (SF-12) |  | Mean VAS pain intensity 5.16 ± 2.90 Significant association between number of exostoses and number of surgeries and between surgical procedures and VAS pain intensity. SF-12 bodily pain not stated separately |  |  | Future studies will focus on the evaluation of sports activity in patients with HME. |
| Hereditary multiple exostosis and pain; Darilek S 2005 | Cross sectional study |  | Mean age 28.2 y, 132 male (45.1%), 161 female (54.9%) number: 293 | Pain interference measured with numeric rating scales and pain drawing pain yes no. Generalized pain defined as pain throughout the body as marked on pain drawing, and not localized to areas with exostoses. Frequency of pain - daily weekly, monthly |  | 246 persons (84%) reported having pain Of those 55.1% had generalized pain and 44.9% localized.  Average pain intensity: 1 person no pain, 56 mild pain, 118 moderate pain, 66 Severe pain  Pain interfered with general activity, sleep, social interactions and with mood. |  | 74 persons reported taking pain medication, 17% a prescription narcotic, 26% non-narcotic prescription, 57% over the counter medication. 155 of 246 participants with pain had seen a pain specialist. | Additional studies are needed to define the causes and characteristics of pain in HME. |
| Use of Pediatric Outcomes Data Collection Instrument to Evaluate Functional Outcomes in Multiple Hereditary Exostoses; De Oliveira NSP 2023 | Cross sectional study | 11 months | 11 children between 2 and 10 y; 23 adolescents between 11 and 18 y ; 21 male, 13 female number: 34 | Paediatric Outcomes Data Collection Instrument (PODCI) Description of pain and clinical examination but no result? |  | Pain/comfort scale mean scores significantly lower than for children and adolescents without musculoskeletal disorders. |  |  | Prospective studies of PODCI with bigger samples focused on assessment of its psychometric properties specifically for MHE paediatric population may be of greater value. |
| **Study information: Title, lead author,  publications year** | **Study design** | **Study duration** | **Patient characteristics (age, gender), number** | **Assessment methods pain, both standardized and study specific** | **Assessment methods fatigue both standardized and study specific** | **Results for pain prevalence, type of pain, pain intensity, duration and pain interference** | **Results on fatigue prevalence, Mean fatigue score (SD) fatigue / vitality instruments, intensity duration** | **Treatment strategies and outcomes** | **Future research targets reported by authors** |
| Ankle Mortise Instability in Multiple Hereditary Exostoses; Ebaugh MP 2022 | Case series |  | mean age at surgery 11.8 y, (range 7-15 y). Mean age at questionnaire 19.0 y +/- 3.6 years. 11 males, 5 females number: 16 patients (19 ankles) | AOFAS ankle hindfoot scores (measuring pain, function and alignment) and short form (SF)-36 scores (measuring physical functioning, emotional health, social well-being, and pain level) Description of pain and clinical examination | SF 36 vitality score | Preoperatively, 11 (57.9%) of 19 ankles were considered to be painful. |  | Thirteen underwent operative intervention. 3 ankles conservative. AOFAS pain: 33.0 +/- 6.7 out of 40 for pain  Mean SF36 vitality score 65,5 Â±20.9 (range 25 - 90). Pain levels improved. | Further research with longer-term follow up is needed to identify the best treatment approach and optimize the management of children with MHE. |
| Vertebral body exostosis and spinal cord compression (Exostoses vertébrales et compression médullaire); El Quessar A 1998 | Case report |  | 13 y, female  number: 1 | Description of pain and clinical examination |  | Bilateral sciatica, predominantly on the right side with and cauda equina compression syndrome Duration: 1 year |  | surgery The patient had a rapid improvement of clinical symptoms |  |
| Two siblings followed up for hereditary multiple exostoses; Erol M 2014 | Case series |  | 8 y, male, 10 y, female number: 2 | Description of pain and clinical examination |  | Generalized pain and swelling in different parts of the body for several years |  |  |  |
| Total knee arthroplasty in patients with multiple hereditary exostoses; Fernandez-Perez SA 2018 | Case report | operation and six months follow up | 67 y, male number: 1 | Western Ontario McMaster Score, Knee Society Knee Score, but not separately results for pain Description of pain and clinical examination |  | Progressive bilateral knee pain for 8 years interfering with walking |  | Surgery: knee prosthesis No results for pain after surgyer |  |
| **Study information: Title, lead author,  publications year** | **Study design** | **Study duration** | **Patient characteristics (age, gender), number** | **Assessment methods pain, both standardized and study specific** | **Assessment methods fatigue both standardized and study specific** | **Results for pain prevalence, type of pain, pain intensity, duration and pain interference** | **Results on fatigue prevalence, Mean fatigue score (SD) fatigue / vitality instruments, intensity duration** | **Treatment strategies and outcomes** | **Future research targets reported by authors** |
| A mountain among molehills: removing an impinging large femoral neck osteochondroma in a man with hereditary multiple exostoses; Fitzgerald CWR 2014 | Case report | Operation and six-week follow-up | 31 y, male number: 1 | Harris Hip score, no separate data for pain Description of pain and clinical examination |  | Increasing right groin pain interfering with physical activity and daily living |  | surgery Pain, distance walked and sitting comfort had considerably improved. |  |
| Hereditary multiple exostoses: a qualitative study exploring families’ and patients’ perceptions of disease impact and self-expressed needs; Fraser M, Porter DE 2000 | Qualitative descriptive study |  | mean age affected female 31 y, range 22 - 40 y (3 participants), male 36 y, range 19 - 55 y (3 participants) number: 13, 6 with MO and 7 unaffected relatives | questionnaire, with both closed and open questions incl. questions on extend and frequency of pain |  | Skeletal and muscular pain, deformities and physical restrictions were amongst the functional effects most frequently cited. |  |  |  |
| A Case Report on Surgical Excision of Intracapsular Osteochondroma of Femur Neck using Mini-Arthrotomy without Hip Dislocation in a Young Female with Hereditary Multiple Exostoses; Ghoti S 2022 | Case report | operation and three years follow-up | 25 y, female number: 1 | Description of pain and clinical examination |  | complaints of pain in the right hip Duration: 1 year difficulty in walking and running |  | Surgery Patient is pain free and able to perform all the daily activities. No recurrence of the lesion on 3-year follow-up |  |
| **Study information: Title, lead author,  publications year** | **Study design** | **Study duration** | **Patient characteristics (age, gender), number** | **Assessment methods pain, both standardized and study specific** | **Assessment methods fatigue both standardized and study specific** | **Results for pain prevalence, type of pain, pain intensity, duration and pain interference** | **Results on fatigue prevalence, Mean fatigue score (SD) fatigue / vitality instruments, intensity duration** | **Treatment strategies and outcomes** | **Future research targets reported by authors** |
| Pain, physical and social functioning, and quality of life in individuals with multiple hereditary exostoses in The Netherlands: a national cohort study; Goud AL 2012 | Cross-sectional study |  | Mean age children 9.4 y (range 1-17 y), mean age adults 39.7 y (range 18-80 y).  Children, 53 boys, 46 girls.  Adults, 75 men, 109 women.  Number: 283 (out of 322 asked, 88%), 184 adults, 99 children | Children and adults: Pain interference measured with numeric rating scales and pain drawings. For adults RAND 36 bodily pain scale There were multiple questions on pain perception, which were based on previous research by Darilek et al. | For adults RAND 36 vitality scale | Pain last thirty days: 62 children ; Adults: 152 adults Adults: RAND 36 Bodily pain scale score significantly lower than three comparison groups. Pain interfered with physical activity and daily living. procedures. | Adults: RAND 36 vitality scale score significantly lower than three comparison groups. Mean RAND36 vitality score 61.3 (SDÂ± 17.6). | Treatment strategies was pain medication, rest and heat application. | More studies with longer follow-up are needed to gain insight into the need for professional support in managing physical and psychosocial problems of patients with multiple hereditary exostoses. |
| Total knee arthroplasty with simultaneous tibial shaft osteotomy in patient with multiple hereditary osteochondromas and multiaxial limb deformity - a case report; Grzelecki D 2020 | Case report | Operation and one year follow up | 65 y, male number: 1 | Description of pain and clinical examination |  | Patient presented with secondary osteoarthritis and severe continual right knee pain. |  | Surgery (Knee prosthesis)  At 6 weeks and 1 year follow-up patient reported no knee pain or gait problems. | Further research is needed in a larger group of patients |
| Hereditary Multiple Exostoses Presenting with Chest Pain in Adolescent Male; Gustafson B 2021 | case report, conference abstract |  | 15 y, male number: 1 | Description of pain and clinical examination |  | months-long history of recurrent left-sided chest pain |  | The decision was made to monitor symptoms and obtain repeat imaging in six months to monitor growth or worsening lung impingement. |  |
| **Study information: Title, lead author,  publications year** | **Study design** | **Study duration** | **Patient characteristics (age, gender), number** | **Assessment methods pain, both standardized and study specific** | **Assessment methods fatigue both standardized and study specific** | **Results for pain prevalence, type of pain, pain intensity, duration and pain interference** | **Results on fatigue prevalence, Mean fatigue score (SD) fatigue / vitality instruments, intensity duration** | **Treatment strategies and outcomes** | **Future research targets reported by authors** |
| Multiple Distal Femoral Osteochondromas Encasing Popliteal Neurovascular Bundle; Habeeb A 2023 | Case report | operation and 6 weeks follow-up | 17 y, male number: 1 | Description of pain and clinical examination |  | Painful lump on the distal left thigh, located medial and just above the knee joint most noticeable on walking Duration 1 year, becoming progressively worse and limiting the ability to walk and participate in sports |  | Surgery At the six-week follow-up. the patient reported an uneventful recovery and full resolution of his symptoms. |  |
| Multiple Hereditary Exostoses Presenting as Painful Shoulder and Knee Masses; Hake T 2020 | Case report |  | 33 y, female number: 1 | Description of pain and clinical examination |  | Chief complaint of left shoulder pain. Several years of progressive, atraumatic shoulder pain. Firm mass at the superior scapular border, lateral shoulder paraesthesia, and a painful right medial knee mass. |  | The patient was referred to orthopaedic oncology for ongoing evaluation, monitoring, and potential resection. |  |
| Multiple hereditary exostoses with spinal cord compression; Johnston CE 1988 | Case report | Intervention and three-year follow-up | 15 y, male number: 1 | Description of pain and clinical examination |  | Duration 3 month |  | Surgery no pain after surgery, after one year and at final follow-up at 18 years |  |
| Total Hip Arthroplasty Using a Polished Tapered Cemented Stem in Hereditary Multiple Exostosis; Kanda A 2016 | Case report | Operation and thirteen-month follow-up | 61 y, male number: 1 | Description of pain and clinical examination |  | The patient had suffered from coxalgia for a long time. |  | Surgery (hip prosthesis) Thirteen months postoperatively the patient had no coxalgia, but the range of motion of the right hip joint was still limited in flexion and abduction. The patient had no limp and no interference with his activities of daily living. |  |
| **Study information: Title, lead author,  publications year** | **Study design** | **Study duration** | **Patient characteristics (age, gender), number** | **Assessment methods pain, both standardized and study specific** | **Assessment methods fatigue both standardized and study specific** | **Results for pain prevalence, type of pain, pain intensity, duration and pain interference** | **Results on fatigue prevalence, Mean fatigue score (SD) fatigue / vitality instruments, intensity duration** | **Treatment strategies and outcomes** | **Future research targets reported by authors** |
| Total hip arthroplasty in hereditary multiple exostoses with secondary osteoarthritis: A case report; Kim WJ 2019 | Case report | operation and two year follow up | 57 y, female number: 1 | Harris Hip score, no separate data for pain Description of pain and clinical examination |  | Severe right hip pain Duration: past 3 years Pain was not severe at the early stage but had gradually progressed, leading to difficulty walking and severe discomfort during daily living. |  | Surgery (total hip arthroplasty) The patient had no limitations of daily activities, and the Harris Hip Score improved from 35 preoperatively to 82 postoperatively at the 2-year follow-up. | Few reports have assessed surgical treatment for HME patients with symptomatic hip lesions, so in our opinion it would be important to acquire as much information as possible through review of the relevant published literature. |
| Fibular lengthening for the management of translational talus instability in hereditary multiple exostoses patients; Lee DY 2014 | Case series |  | Mean age at surgery 14.4 y (range 8.8 to 19.3 y), 2 females, 7 males number: 9 patients (twelve ankles) | The AOFAS ankle hindfoot score was recorded preoperatively and at the latest follow-up. |  | Ankle pain after physical exercise AOFAS ankle-hindfoot pain component preoperative 30.8/40 |  | Surgery Improvement of AOFAS ankle-hindfoot score was mainly attributed to improvement in pain component and improvement in activity limitation component. |  |
| Daughter and mother diagnosed with hereditary multiple exostoses: A case report and a review of the literature; Marginean CO 2017 | Case report and non-systematic literature review |  | 5 y, female number: 1 | Description of pain and clinical examination |  | inferior limb pain, painful oedema of the right index finger Inferior limb: approximately 1 year,  painful oedema of the right index finger: last month |  | Monitoring once a year The radiological aspect at 6-month follow-up did not reveal any additional modifications. No report on pain status. |  |
| Functional Impairment of Hip Joint and Activities of Daily Living Failure in Patients with Multiple Hereditary Exostoses; Matsumoto K 2022 | Cross sectional study | One year | Median age 25.6 y (range 7-79 y). men 40 hips, women 38 hips 39 patients (78 hips) | Japanese Orthopaedic association Hip score: Including pain score, range of motion, ability to walk and activities of daily living. Perfect JOA hip score was 100. questionnaire on age, ethnicity, medication history and disease history. |  | 8 patients experienced pain in their hip joints.  The average JOA pain score was 38.1 ± 5.3. 20% of patients had hip pain, and half of them had some degree of ADL failure. |  |  | further studies are needed to generate statistically strong evidence. |
| **Study information: Title, lead author,  publications year** | **Study design** | **Study duration** | **Patient characteristics (age, gender), number** | **Assessment methods pain, both standardized and study specific** | **Assessment methods fatigue both standardized and study specific** | **Results for pain prevalence, type of pain, pain intensity, duration and pain interference** | **Results on fatigue prevalence, Mean fatigue score (SD) fatigue / vitality instruments, intensity duration** | **Treatment strategies and outcomes** | **Future research targets reported by authors** |
| Chest pain caused by multiple exostoses of the ribs: A case report and a review of literature; Mazza D 2017 | Case report and non-systematic literature review | Operation and 1 year follow-up | 16 y, male number: 1 | Description of pain and clinical examination |  | left-sided chest pain pain exacerbated by physical activity and cough |  | Surgery At the last 1-year follow-up, patient was very satisfied, and no signs of recurrence or major complication had occurred. No report on post- surgery pain |  |
| Spinal cord stimulation for treatment of the pain associated with hereditary multiple osteochondromas; Mirpuri RG 2015 | Case report | approximately 18 months | 65 y, female number: 1 | Numeric pain rating scale 0-10, with 0 reflecting no pain, 1-3 reflecting mild pain and mild interference on activities of daily living (ADL), 4-6 reflecting moderate pain and moderate interference on ADL, and 7-10 reflecting severe pain and inability to perform ADL. |  | Back and bilateral leg pain, characterized as constant, heavy, and aching with an intermittent stabbing component that radiated from the back into her lower extremities. Isolated aching pain also in the left hip, knees, and ankles. Duration: 4 years Exacerbating factors included walking, exercise, spinal flexion, and rotation |  | Lumbar epidural steroid  injections, ischial bursa injections, and bilateral piriformis injections. Morfins, neuropathic medications without enough pain relief Spinal Cord Stimulation After 5 days: 70% - 80% improvement in pain and 85% reduction in oxycodone IR intake.  On 8-week follow-up another 30% reduction in pain compared to last  visit's baseline, complete discontinuation of methadone, and increased ADL. At 6-month follow-up, the patient reported continued relief. | Further research should be considered in treating neoplastic-mediated pain that has been resistant to both pharmacological and surgical modalities with SCS |
| Autologous Fat Grafting as a Last Resort for Unsustainable Pain in a Woman with Multiple Osteochondromas; Negenborn VL 2017 | Case report | approximately two years follow-up | 48 y, female number: 1 | Visual analogue scale for pain, 0-10 Description of pain and clinical examination |  | Painful and residual, though very small, osteochondroma originating from the left scapula, and persistent pain from symptomatic osteochondromas at the 5th digit of the left foot and in the trochanter region VAS score at presentation was 8. |  | Local injection therapy with corticosteroids, and analgesics, surgery - minimal effect for a limited period.  Autologous Fat Grafting  After treatment reduction in pain (VAS 3), no pain medication needed. Only partial recurrence of the pain, which was also not as severe as before the treatment. | Future studies including more patients in a controlled setting are necessary to confirm our results. |
| **Study information: Title, lead author,  publications year** | **Study design** | **Study duration** | **Patient characteristics (age, gender), number** | **Assessment methods pain, both standardized and study specific** | **Assessment methods fatigue both standardized and study specific** | **Results for pain prevalence, type of pain, pain intensity, duration and pain interference** | **Results on fatigue prevalence, Mean fatigue score (SD) fatigue / vitality instruments, intensity duration** | **Treatment strategies and outcomes** | **Future research targets reported by authors** |
| Total Hip Arthroplasty with a Revision Stem in Hereditary Multiple Exostoses with Secondary Osteoarthritis; Negri ME 2022 | Case report | surgery and 7 years follow-up | 42 y, female number: 1 | Haris Hip score, but no separate data on pain Description of pain and clinical examination |  | left coxalgia Duration: 2 years severe pain during weight bearing and limitations on his daily activities |  | Surgery (hip prosthesis) Pain-free 7 years later; Harris hip score 100 (from 75) |  |
| Evaluation of the forearm in untreated adult subjects with multiple hereditary osteochondromatosis; Noonan KJ 2002 | Cross-sectional study |  | Mean age 42 y (range 20-80 y), 22 male, 17 female number: 39, 77 upper extremities | Visual Analog Scale (VAS) 0-10, pain and functional assessment scale (Stanton and Hansen). Questions on demographics and any surgery on the upper extremities, limitations in their career choice or ability to perform certain recreational activities because of forearm or wrist deformities. |  | 77% reported that they were free of pain and major limitations in the arms. Mean VAS pain score 2.3 (range 0 to 9). 47 (61%) had no limitations in activities due to pain. 2 had pain for which they took medication. 9% had pain in forearm, elbow, or wrist which limited activities. |  |  | a future comparison of the findings in treated patients with our findings in untreated patients will accurately document the positive or negative effects of treatment on the natural history of the disease |
| Natural history of multiple hereditary osteochondromatosis of the lower extremity and ankle; Noonan KJ 2002 | Cross sectional study |  | Mean age 42 y (range 20-80 y), 17 males, 21 females number: 38 | Incidence and severity of ankle pain were recorded on a visual analogue scale, 0 no pain and 10 defined as the most severe pain ever experienced. In addition, subjects were asked to rate their ankles using a modification of the Stanton and Hansen functional status scale Questionnaire on medical and surgical history and functional limitations related to ankle involvement |  | 8 had pain in ankle, which limited activities. 4 had pain in ankle for which they took medication. 7 had pain at least once a week, 2 had constant ankle pain.  Average ankle pain score was 2.2 (range 0 - 8). 19 reported having occasional pain when walking. |  | None of the subjects in this study had undergone a reconstructive procedure to improve alignment, such as an osteotomy or leg lengthening. Twelve subjects (32%) had removal of osteochondromas of the lower extremity. |  |
| **Study information: Title, lead author,  publications year** | **Study design** | **Study duration** | **Patient characteristics (age, gender), number** | **Assessment methods pain, both standardized and study specific** | **Assessment methods fatigue both standardized and study specific** | **Results for pain prevalence, type of pain, pain intensity, duration and pain interference** | **Results on fatigue prevalence, Mean fatigue score (SD) fatigue / vitality instruments, intensity duration** | **Treatment strategies and outcomes** | **Future research targets reported by authors** |
| Management of nerve compression in multiple hereditary exostoses: a report of two cases and review of the literature; Payne R 2016 | Case series and non-systematic literature review | 3 - 4 years for each patient | Patient one: 16 y, female. patient two: 15 y, female number: 2 | Description of pain and clinical examination |  | P1: age 13- musculoskeletal pain, age 15- femoral nerve, neuropathic pain.  P2: at age 10 musculoskeletal pain, at age 14 significant pain in distal left extremity, age 15 chronic dislocation of left radial head with surrounding pain, age 15 right common fibular nerve and right tibial nerve pain |  | Surgery improvement/ relief in pain after surgery for some surgeries, but not alle, for both patients |  |
| Pes Anserinus Syndrome Caused by Osteochondroma in Paediatrics: A Case Series Study; Sakamoto A 2017 | Case series | 6-month follow-up on patient 1, 24 months for patient 2 | case no 4: 12 y, female, case number 5: 15 y, female number: 2 | Description of pain and clinical examination |  | Case 4: difficulty in fully flexing the right knee because of pain 12 years old. No pain while walking. symptoms lasted for 2-3 years.  Case 5: Presented with pain on the medial aspect of the right leg when she changed clothes while sitting on her buttocks and flexing the knee 15 years old,  Pain had been present for six. |  | Case 4: Surgery Case 5: No treatment Case 4: pain free,  Case 5: Pain appeared occasionally, especially on flexing the knee, but did not disturb activities of daily living. The pain did not require medication, but the symptoms still persisted at the time of the two-year follow-up. |  |
| **Study information: Title, lead author,  publications year** | **Study design** | **Study duration** | **Patient characteristics (age, gender), number** | **Assessment methods pain, both standardized and study specific** | **Assessment methods fatigue both standardized and study specific** | **Results for pain prevalence, type of pain, pain intensity, duration and pain interference** | **Results on fatigue prevalence, Mean fatigue score (SD) fatigue / vitality instruments, intensity duration** | **Treatment strategies and outcomes** | **Future research targets reported by authors** |
| Total Knee Arthroplasty With Patient-Specific Instrumentation to Correct Severe Valgus Deformity in a Patient With Hereditary Multiple Exostoses; Sasaki U 2022 | Case report | Intervention and one year follow up | 62 y, female number: 1 | Knee Society Score, no separate data for pain Description of pain and clinical examination |  | Considerable pain during motion and tenderness in the left medial joint space (knee). Duration: more than 8 years The preoperative Knee Society Scores were 8 (objective knee indicators), 4 (symptoms),16 (patient satisfaction), 12 (patient expectations), and 39 (functional activities). |  | oral medication and therapeutic injections - pain remained, surgery (knee prosthesis) Postoperatively the patient had no pain in her left knee while ambulating, and her walking distance progressed to >2000 m at the latest follow-up (1 year postoperatively). | larger-scale, long-term investigations are necessary |
| Modified Ilizarov technique for the treatment of forearm deformities in multiple cartilaginous exostoses: case series and literature review; Song SH 2013 | Case series with retrospective chart review and cross-sectional questionnaire +non-systematic literature review |  | mean age 17.4 y, range 4 to 36 y at time of surgery, 9 males, 7 females number: 16 patients (23 forearms) | Questionnaire with evaluation of outcomes with respect to pain, activities of daily living, and appearance of the operated forearm. Pain and restriction of activities were classified in four levels: none, mild, moderate, severe, appearance as satisfactory or unsatisfactory |  | Pain assessment pre-operative state: 1=none, 6=mild, 7=moderate, 2=severe. Five patients presented with mild pain when performing any strenuous activity, whereas the other 11 patients had no pain when performing any strenuous activity at the time of final follow-up |  | Surgery  post-op 0 severe pain, 0 moderate, 5 mild, 11 none |  |
| **Study information: Title, lead author,  publications year** | **Study design** | **Study duration** | **Patient characteristics (age, gender), number** | **Assessment methods pain, both standardized and study specific** | **Assessment methods fatigue both standardized and study specific** | **Results for pain prevalence, type of pain, pain intensity, duration and pain interference** | **Results on fatigue prevalence, Mean fatigue score (SD) fatigue / vitality instruments, intensity duration** | **Treatment strategies and outcomes** | **Future research targets reported by authors** |
| One-stage surgical excision of a huge bilateral multiple osteochondroma of the hip: a case report; Taheriazam A 2017 | Case report |  | 25 y, male number: 1 | Description of pain and clinical examination |  | Severe swelling and pain in both hips for the past 12 months. Daily activities significantly restricted due to the restricted movement of the hip joint. Problems in walking and other motions related to the hip joints activities. In the recent 6 months before admission, his chief complaint was in sitting. |  | Surgery no symptoms after surgery |  |
| Management of forearm deformities with ulnar shortening more than 15 mm caused by hereditary multiple osteochondromas; Tang ZW 2013 | Case series | 5 years | average age 9.2 y (range 4-15 y), 6 female, 8 male number: 14 | Pain and restriction of activities were classified into four categories (none, mild, moderate) pain and restriction of activities were classified into four categories (none, mild (pain during activity), moderate (pain at rest), severe) |  | At initial evaluation None had severe pain, 10% had moderate pain, 15% mild pain and 20% no pain. |  | Surgery During the mean 43.1 months follow-up, four patients had mild pain, and two patients had mild restriction of daily activities. |  |
| Selective computed tomography-guided perisciatic injection as a diagnostic tool in multiple hereditary exostoses; Tenenbaum S 2012 | Case report | Intervention and one year follow-up | 58 y, male number: 1 | Description of pain and clinical examination |  | right sciatic pain Duration: Last five years he was unable to sit for a few minutes; therefore, he spent all waking hours standing, walking, or lying down. |  | Test with perisciatic injection - pain relief Surgery pain free, returned to work and sat without discomfort at 1 year follow-up |  |
| **Study information: Title, lead author,  publications year** | **Study design** | **Study duration** | **Patient characteristics (age, gender), number** | **Assessment methods pain, both standardized and study specific** | **Assessment methods fatigue both standardized and study specific** | **Results for pain prevalence, type of pain, pain intensity, duration and pain interference** | **Results on fatigue prevalence, Mean fatigue score (SD) fatigue / vitality instruments, intensity duration** | **Treatment strategies and outcomes** | **Future research targets reported by authors** |
| Diaphyseal Aclasis With Pes Anserinus Syndrome; Tiwari V 2021 | Case report |  | 20 y, male number: 1 | Visual analogue scale VAS post op Description of pain and clinical examination |  | right upper leg pain, with no relief after taking analgesics.  Duration: Over six months |  | Surgery pain-free, VAS 0, at one year follow-up |  |
| Lower limb osteotomies for joint realignement in a patient with hereditary multiple exostoses and symptomatic bone deformity: A case report; Urbani E 2019 | Case report, conference abstract |  | 54 y, male number: 1 | Description of pain and clinical examination |  | pain right ankle joint, due to valgus malalignment, medial compartment osteoarthritis of the left knee Duration: 7 years |  | Surgery Pain relief after both surgeries |  |
| Bilateral total hip arthroplasty in a young man with hereditary multiple exostoses; Vaishya R 2015 | Case report | 12-month follow-up | 27 y, male number: 1 | Haris Hip score, but no separate data on pain Description of pain and clinical examination. Patient's qualitative assessment |  | bilateral hip pain. It first developed in the left hip and subsequently also affected the right hip. It was insidious in onset and mild in intensity initially, but gradually progressed to severe and constant pain. Duration: 1.5 years The pain was severe enough to prevent him from walking and performing activities of daily living independently. |  | Surgery (hip prosthesis) At follow-up, after 12 months both hips were found stable, and the osteotomy on the right side had healed. The patient was mobilising with full weight bearing. Patients’ perspective: I was feeling hopeless due to the complex nature of my problem and as no definitive treatment was offered to me. I was suffering with severe agonising pain in the hips. However, after bilateral hip surgery, I felt as if a new lease of life had been given to me. |  |
| Cervical Osteochondroma Causing Myelopathy in Adults: Management Considerations and Literature Review; Veeravagu A 2017 | Case series and non-systematic literature review |  | Patient 1: 22 y, male, Pat 2: 20 y, female number: 2, but pain only described for 1 of them (pat 1) | Description of pain and clinical examination |  | Pat 1: intermittent right-sided neck pain associated with limited range of motion, as well as right arm weakness and numbness Duration: 1: 5 month |  | Pat 1: Surgery. At 3month follow-up visit, patient reported only some residual weakness in his right first and second digit, but he was otherwise back to work and feeling well. No information on pain. |  |
| **Study information: Title, lead author,  publications year** | **Study design** | **Study duration** | **Patient characteristics (age, gender), number** | **Assessment methods pain, both standardized and study specific** | **Assessment methods fatigue both standardized and study specific** | **Results for pain prevalence, type of pain, pain intensity, duration and pain interference** | **Results on fatigue prevalence, Mean fatigue score (SD) fatigue / vitality instruments, intensity duration** | **Treatment strategies and outcomes** | **Future research targets reported by authors** |
| Bilateral ischiofemoral impingement in a patient with hereditary multiple exostoses; Viala P 2012 | Case report | follow-up 6 month after surgery | 37 y, female number: 1 | Description of pain and clinical examination |  | left hip pain motion was limited and painful: pain was increased in abduction, external rotation, and flexion. Adduction against resistance was also painful.  mild to moderate at rest and increased when walking Duration: 2 years |  | Surgery Six months postoperatively, left hip pain was improved, appearing only after walking long distances. |  |
| A 40-Year-Old Male Presenting with Hereditary Multiple Exostosis: Management and Considerations; Wells M 2019 | Case report |  | 40 years, male number:1 | Probably Visual Analogue scale, pain 7/10 reported in results, but VAS not mentioned Clinical description |  | chronic left knee pain VAS 7/10 Pain worse with movements. The patient has chronic pain elsewhere, which is being closely monitored. |  | Surgery (knee prosthesis), manipulation under anaesthesia follower by 6 weeks of physical therapy to improve range of motion. Arthroplasty helped relieve the patients pain, however, did not result in significant improvement of active and passive ROM. |  |
| **Study information: Title, lead author,  publications year** | **Study design** | **Study duration** | **Patient characteristics (age, gender), number** | **Assessment methods pain, both standardized and study specific** | **Assessment methods fatigue both standardized and study specific** | **Results for pain prevalence, type of pain, pain intensity, duration and pain interference** | **Results on fatigue prevalence, Mean fatigue score (SD) fatigue / vitality instruments, intensity duration** | **Treatment strategies and outcomes** | **Future research targets reported by authors** |
| The Impact of Isolated Versus Multiple Osteochondromas: Analysis of the CoULD Registry; Wessel LE 2022 | Cross-sectional study, retrospective registry study | Patients enrolled from 2014 - 2021 | Mean age 9.9 y (range 2.0- 17.9 y), 61 males, 38 females, no separate data for MO group Number: 99 of these 73 with MO | Patient-Reported Outcome Measurement Information System (PROMIS): (Global Functioning and UE function, Pain, Depression, Anxiety, and Peer relations), Paediatric Outcomes Data Collection Instrument (PODCI): (UE function, Transfers and Mobility, Sports and Physical Functioning, Pain/Comfort, Happiness, and Global Functioning). |  | Mean Pain/ comfort PODCI scores worse than for population norms, and lower in patients with MO than solitaire osteochondromas: Association between presence of multiple lesions and worse PROMIS pain interference scores as. Significantly more pain interference in MO patients |  |  | PROs scores in our study were collected at patient enrolment and, as such, do not reflect the impact of surgery on the cohort. Whether surgical intervention improves these PROs is an area of future interest and requires further investigation. |
| Multiple osteochondromas of the cervical spine, a potential cause of radiculopathy in the elderly: A case report and review of literature; Yudistra A 2020 | Case report and non-systematic review | Follow-up after surgery 18 month | 76 y, female number: 1 | Description of pain and clinical examination |  | pain and numbness of the left suboccipital and preauricular region Duration: 6 months |  | Surgery Pain decreased seven days after surgery. 18 months after surgery - moderate neck pain. |  |
| Osteochondroma and Spinal Cord Compression in a Patient With Hereditary Multiple Exostoses: A Case Report; Zoboski RJ 2017 | Case report | Intervention and two year follow up | 31 years, female number: 1 | Description of pain and clinical examination |  | past few years, pain present since age 16 |  | Surgery Postoperative pain required the use of opioids, continued for approximately 3 months after the surgery. 6 months of physical therapy, overall subjective improvement of 90%. Postoperative thoracic pain returned after 2 years. The patient responded favourably to a brief course of physical therapy. |  |
