# Supplementary material for: Chronic pain and fatigue in multiple osteochondroma and Ollier disease, a systematic review
Source: BMC Musculoskelet Disord. 2026 May 26;27:624. doi: 10.1186/s12891-026-10025-6 (PMC13393944; doi:10.1186/s12891-026-10025-6)
Supplement: Supplementary file 3 — Additional file 3. [file 12891_2026_10025_MOESM3_ESM.docx]

| **Supplementary table 3 Quality assessment of included articles on multiple osteochondromas (MO) and Olliers disease (OD)** | | | | | | | | | | | |
| --- | --- | --- | --- | --- | --- | --- | --- | --- | --- | --- | --- |
| **Title, year, first author**  **Case studies** | **1. Were patient’s demographic characteristics clearly described?** | **2. Was the patient’s history clearly described and presented as a timeline?** | **3. Was the current clinical condition of the patient on presentation clearly described?** | **4. Were diagnostic tests or assessment methods and the results clearly described?** | **5. Was the intervention(s) or treatment procedure(s) clearly described?** | **6. Was the post-intervention clinical condition clearly described?** | **7. Were adverse events (harms) or unanticipated events identified and described?** | **8. Does the case report provide takeaway lessons?** | **Number yes and n/a** | **Quality** | **Contribution to knowledge om chronic pain and fatigue** |
| Exostose vertébrale et compression médullaire, 1998, El Quessar | yes | no | no | yes | no | no | no | no | 2 | Low | No use of standardized pain measurement. Minimal information about pain in spinal osteochondroma. |
| Lower limb osteotomies for joint realignment in a patient with hereditary multiple exostoses and symptomatic bone deformity: a case report, 2019, Urbani | yes | no | no | no | yes | yes | no | no | 3 | Low | Conference abstract with limited data. Some information on knee deformity and pain. No validated pain measures used. |
| Joint pain in hereditary multiple exostoses, 2015, Bruce | no | no | no | no | n/a | n/a | n/a | no | 3 | Low | Limited contribution to knowledge in pain. |
| Diaphyseal Aclasis With Pes Anserinus Syndrome, 2021, Tiwari | yes | yes | no | yes | yes | yes | no | unclear | 5 | moderate | Fair contribution to knowledge on, diagnostic methods and treatment of osteochondroma and pain in MO. Reports patient's evaluation of pain pre- and post-surgery. VAS scale only post-surgery. |
| A rare case of Enchondromatosis of the knees and hands with involvement of Hoffa's fat pad and peri-articular soft-tissues. 2013, Sutera | yes | yes | no | no | n/a | n/a | n/a | no | 5 | moderate | Gives little contribution to knowledge on enchondromas and pain, description of symptoms, no treatment described. |
| Buttock Pain and Sciatica Caused by a Femoral Osteochondroma, 2017, Akinyemi | no | no | yes | yes | yes | yes | no | yes | 5 | moderate | Shows that surgery can be a good method to reduce pain when pain is clearly related to exostosis, good use of validated instruments pre - and post operative. |
| Bilateral scapular osteochondroma in Multiple Hereditary Exostosis patient presented with bilateral shoulder pain treated with arthroscopic and open excision: Case report, 2021, Alshayhan | no | no | yes | no | yes | yes | yes | yes | 5 | moderate | Compares arthroscopy and open surgery on same patient. Fair contribution on scapular osteochondroma in MO. |
| Rehabilitation experience in a case of Ollier’s disease, 2003, Formis | yes | no | yes | yes | no | yes | no | yes | 5 | moderate | Important contribution to knowledge on pain, fatigue (vitality) and rehabilitation in patient with OD. Standardized measured used pre- and post-intervention showing improvement in function, quality of life, pain and vitality. |
| Modified Technique of Single-Bone Forearm in the Treatment of Deformities, 2022, Benameur | no | no | yes | yes | yes | yes | yes | yes | 6 | moderate | Good use of validated instruments pre - and post operative on pain and function. |
| A mountain among molehills: removing an impinging large femoral neck osteochondroma in a man with hereditary multiple exostoses, 2014, Fitzgerald | yes | no | yes | yes | yes | yes | no | yes | 6 | moderate | Shows that surgery can be a good method to reduce pain when pain is clearly related to exostosis. No separate data for pain. |
| Three different methods for treating multiple enchondromatosis in one hand, 2015, Lu | yes | no | yes | yes | yes | yes | no | yes | 6 | moderate | Shows that surgery can be a good method to reduce pain when pain is clearly related to OD. No standardized measurements of pain. |
| spinal cord stimulation for treatment of the pain associated with hereditary multiple osteochondromas, 2015, Mirpuri | yes | no | yes | yes | yes | yes | no | yes | 6 | moderate | Describes successful use of SCS in a patient with MO, but both failed back surgery, MO and possibly other factors may play a role in patient’s pain. Detailed description on validated measures for pain and ADL pre, post treatment and at 6 months follow-up. |
| A Case Report on Surgical Excision of Intracapsular Osteochondroma of Femur Neck using Mini-Arthrotomy without Hip Dislocation in a Young Female with Hereditary Multiple Exostoses, 2022, Ghoti | yes | no | yes | yes | yes | yes | no | yes | 6 | moderate | Some contribution to knowledge on intracapsular osteochondroma of femur neck and pain in MO. Reports patient's evaluation of pain pre- and post-surgery. No validated pain measures. |
| Local design and manufacturing of patient-specific implant using Anatomage Medical Design Studio software: proof of concept - Botswana's 1st case report, 2023, Nkhwa | yes | no | yes | yes | yes | yes | no | yes | 6 | moderate | Some contribution to knowledge on OD and pain. Focus on use of 3d print and implant making in Botswana and outcome of correction of limb length differences, Reports patient’s evaluation of pain pre- and post-surgery with validated pain scale. |
| Multiple Hereditary Exostoses Presenting as Painful Shoulder and Knee Masses, 2020, Hake | yes | no | yes | yes | n/a | n/a | n/a | no | 6 | moderate | Limited contribution to knowledge on osteochondroma and pain in MO. no standardized measures used for pain intensity or interference. |
| Total knee arthroplasty with simultaneous tibial shaft osteotomy in patient with multiple hereditary osteochondromas and multiaxial limb deformity - a case report, 2020, Grzelecki | yes | no | yes | yes | yes | yes | yes | yes | 7 | high | Shows that surgery can be a good method to reduce pain when pain is clearly related to exostosis. No standardized measurements of pain. |
| Multiple Distal Femoral Osteochondromas Encasing Popliteal Neurovascular Bundle, 2023, Habeeb | yes | no | yes | yes | yes | yes | yes | yes | 7 | high | Some contribution to knowledge on osteochondroma pain in MO. Reports patient's evaluation of pain pre- and post-surgery. No validated pain measures. |
| Total Hip Arthroplasty with a Revision Stem in Hereditary Multiple Exostoses with Secondary Osteoarthritis, 2022, Negri | yes | no | yes | yes | yes | yes | yes | yes | 7 | high | Some contribution to knowledge on osteochondroma pain in MO. Reports patient's evaluation of pain pre- and post-surgery. Use of Harris Hip Score, but no separate data for pain. |
| Timing of forearm deformity correction in a child with multiple hereditary exostosis, 2014, Beutel | no | yes | yes | no | yes | no | yes | yes | 5 | moderate | Limited contribution to knowledge on pain, focus is on function. |
| Subacromial impingement syndrome in hereditary multiple exostoses, 1986, Craig | no | yes | yes | no | yes | yes | no | yes | 5 | moderate | Shows that surgery can be a good method to reduce pain when pain is clearly related to exostosis. No standardized measurements of pain. |
| Bilateral ischiofemoral impingement in a patient with hereditary multiple exostoses, 2012, Viala | yes | yes | yes | yes | yes | no | no | yes | 6 | moderate | Some contribution to knowledge on, ischiofemoral impingement and pain in MO. No validated pain measures used. |
| Osteochondroma and Spinal Cord Compression in a Patient With Hereditary Multiple Exostoses: A Case Report, 2016, Zoboski | yes | yes | yes | yes | no | yes | no | yes | 6 | moderate | Some contribution to knowledge on, spinal osteochondroma and pain in MO. Reports patient's evaluation of pain pre- and post-surgery. no validated pain measures |
| Multiple enchondromatosis: a case report, 2002, Benbouazza | no | yes | yes | no | n/a | n/a | n/a | yes | 6 | moderate | Describes that chronic pain is a problem in OD. |
| Enchondromatosis affecting the foot: A case report, 2021, Edwards | yes | yes | yes | yes | yes | yes | no | yes | 7 | high | Describes a pathological fracture in OD with pain over long time which is cured with surgery. No use of standardized pain measurement. |
| Multiple enchondromatosis: a case report and review of the literature, 1975, Jacobs | yes | yes | yes | yes | yes | no | yes | yes | 7 | high | Shows that surgery can be a good method to reduce pain when pain is clearly related to enchondromas. no standardized measurements of pain. |
| Multiple hereditary exostoses with spinal cord compression, 1988, Johnston | yes | yes | yes | yes | yes | yes | no | yes | 7 | high | Shows that surgery can be a good method to reduce pain when pain is clearly related to exostosis. No standardized measurements of pain. |
| One-stage surgical excision of a huge bilateral multiple osteochondroma of the hip: a case report, 2017, Taheriazam | yes | yes | yes | yes | yes | no | yes | yes | 7 | high | Fair contribution to knowledge on hip osteochondroma and pain in MO. Reports patient's evaluation of pain pre- and post-surgery. No validated pain measures used. |
| Total hip arthroplasty in hereditary multiple exostoses with secondary osteoarthritis: A case report, 2019, Kim | yes | yes | yes | yes | yes | yes | no | yes | 7 | high | Shows that surgery can be a good method to reduce pain when pain is clearly related to exostosis. No separate measurements of pain. |
| Chest pain caused by multiple exostoses of the ribs: A case report and a review of literature, 2017, Mazza | no | yes | yes | yes | yes | yes | yes | yes | 7 | high | Describes the rare existence of exostosis in rib that gives chest pain |
| Selective computed tomography-guided perisciatic injection as a diagnostic tool in multiple hereditary exostoses, 2012, Tenenbaum | yes | yes | yes | yes | yes | yes | no | yes | 7 | high | Contribution to knowledge on, diagnostic methods and treatment of sciatic pain in MO. Reports patient's evaluation of pain pre- and post-surgery. no validated pain measures used. |
| A 40-Year-Old Male Presenting with Hereditary Multiple Exostosis: Management and Considerations, 2019, Wells | yes | yes | yes | yes | yes | yes | no | yes | 7 | high | Some contribution to knowledge on, knee arthroplasty and pain in MO. Reports patient's evaluation of pain pre- and post-surgery. no validated pain measures. |
| Daughter and mother diagnosed with hereditary multiple exostoses, 2016, Marginean | yes | yes | yes | yes | n/a | n/a | n/a | no | 7 | high | Limited contribution to knowledge on osteochondroma and pain in MO. no information on pain status at follow up. no standardized measures used for pain intensity or interference. |
| Hereditary Multiple Exostoses Presenting with Chest Pain in Adolescent Male, 2021, Gustafson | yes | yes | yes | yes | n/a | n/a | n/a | yes | 8 | high | Give some knowledge on chest pain as only symptom of exostoses in MO. This is a conference abstract and therefore give limited data. Minimal information on pain and development of pain symptoms. no standardized pain measures used |
| Total Hip Arthroplasty Using a Polished Tapered Cemented Stem in Hereditary Multiple Exostosis, 2016, Kanda | yes | yes | yes | yes | yes | yes | yes | yes | 8 | high | Shows that surgery can be a good method to reduce pain when pain is clearly related to exostosis. No separate data on pain. |
| Autologous Fat Grafting as a Last Resort for Unsustainable Pain in a Woman with Multiple Osteochondromas, 2017, Negenborn | yes | yes | yes | yes | yes | yes | yes | yes | 8 | high | Describes the method of autologous fat grafting as treatment for chronic pain in one patient with MO. Validated pain measures of pain used pre and post treatment. |
| Total Knee Arthroplasty With Patient-Specific Instrumentation to Correct Severe Valgus Deformity in a Patient With Hereditary Multiple Exostoses, 2022, Sasaki | yes | yes | yes | yes | yes | yes | yes | yes | 8 | high | Contribution to knowledge on knee arthroplasty and pain in MO. Reports patient's evaluation of pain pre-, post-surgery and at 12 months follow-up. Also, postoperative knee scores showed improvement in symptoms. |
| Bilateral total hip arthroplasty in a young man with hereditary multiple exostoses, 2014, Vaishya | yes | yes | yes | yes | yes | yes | yes | yes | 8 | high | contribution to knowledge on, treatment of hip osteochondroma and pain in MO. Reports patient's evaluation of pain pre- and post-surgery. |
| Multiple osteochondromas of the cervical spine, a potential cause of radiculopathy in the elderly: A case report and review of literature, 2020, Yudistira | yes | yes | yes | yes | yes | yes | yes | yes | 8 | high | Some contribution to knowledge on, spinal osteochondroma and pain in MO. Little details on patient's evaluation of pain pre- and post-surgery. No validated pain measures |
| Total knee arthroplasty in patients with multiple hereditary exostoses, 2018, Fernandez-Perez | yes | yes | yes | yes | yes | yes | yes | yes | 8 | high | Shows that surgery can be a good method to reduce pain when pain is clearly related to exostosis. No separate data for pain. |

| **Title, year, first author**  **Case series** | **1. Were there clear criteria for inclusion in the case series?** | **2. Was the condition measured in a standard, reliable way for all participants included in the case series?** | **3. Were valid methods used for identification of the condition for all participants included in the case series?** | **4. Did the case series have consecutive inclusion of participants?** | **5.Did the case series have complete inclusion of participants?** | **6. Was there clear reporting of the demographics of the participants in the study?** | **7.Was there clear reporting of clinical information of the participants?** | **8. Were the outcomes or follow up results of cases clearly reported?** | **9. Was there clear reporting of the presenting site(s)/clinic(s) demographic information?** | **10. Was the statistical analysis appropriate?** | **Number yes and not applicable** | **Quality** | **Contribution to knowledge om chronic pain and fatigue** |
| --- | --- | --- | --- | --- | --- | --- | --- | --- | --- | --- | --- | --- | --- |
| Management of nerve compression in multiple hereditary exostoses: a report of two cases and review of the literature, 2016, Payne | no | yes | yes | no | unclear | yes | yes | yes | no | n/a | 6 | moderate | Gives fair knowledge on treatment of nerve compression and pain in MO-Patients. Evaluation of pain pre and post treatment. No validated measures used. |
| Pes Anserinus Syndrome Caused by Osteochondroma in Paediatrics: A Case Series Study, 2017, Sakamoto | no | yes | yes | no | no | yes | yes | yes | no | n/a | 6 | moderate | Gives some knowledge on pes anserinus syndrome and pain in 2 MO patients. Patients’ evaluation of paint pre and post treatment. No validated measures used. |
| Cervical Osteochondroma Causing Myelopathy in Adults: Management Considerations and Literature Review, 2016, Veeravagu | no | yes | yes | unclear | unclear | yes | yes | yes | no | n/a | 6 | moderate | Limited contribution to knowledge on pain and cervical osteochondroma in one of the cases. No standardized pain measures given. No post operative data on pain. |
| Bisphosphonates for Pain Management in Children With Benign Cartilage Tumors, 2012, Winston | no | yes | yes | unclear | unclear | yes | yes | yes | unclear | n/a | 6 | moderate | Contributes to knowledge on treatment of pain with bisphosphonates in patients with Ollier- disease. Good description of effect of pain both with validated pain scales and child and parents’ personal evaluation on of pain interference pre and post intervention. |
| Two siblings followed up for hereditary multiple exostoses, 2013, Erol | no | yes | yes | yes | yes | yes | yes | no | no | n/a | 7 | moderate | Limited knowledge on pain in two siblings with MO. No standardized pain measures given. no follow-up data on pain. |
| Ankle Mortise Instability in Multiple Hereditary Exostoses, 2022, Ebaugh | yes | yes | yes | yes | yes | yes | yes | yes | no | yes | 9 | high | Gives fair knowledge on treatment of ankle deformities in patients with MO. Also, some information on pain. However standardized function and QoL measures including pain scales, were only used post intervention. |
| Effect of Distal Ulna Osteochondroma Excision and Distal Ulnar Tether Release on Forearm Deformity in Preadolescent Patients With Multiple Hereditary Exostosis, 2020, Belyea | yes | yes | yes | yes | no | yes | yes | yes | yes | yes | 9 | high | Gives fair knowledge on treatment of forearm deformities in patients with MO. Also, some information on pain. |
| Modified Ilizarov technique for the treatment of forearm deformities in multiple cartilaginous exostoses: case series and literature review, 2012, Song | yes | yes | yes | yes | yes | yes | yes | yes | no | yes | 9 | high | Contributes to knowledge on treatment of forearm deformities and pain in patients with MO. Study- specific, structured assessment methods used for pain and activities of daily living pre and postoperatively. |
| Gradual ulnar lengthening in children with multiple exostoses and radial head dislocation: results at skeletal maturity, 2016, D'Ambrosi | yes | yes | yes | yes | yes | yes | yes | yes | no | yes | 9 | high | Contributes to knowledge on treatment of forearm deformities and pain in patients with MO. Standardized pain, function and activity measures pre and post intervention. |
| Fibular Lengthening for the Management of Translational Talus Instability in Hereditary Multiple Exostoses Patients, 2014, Lee | yes | yes | yes | yes | yes | yes | yes | yes | no | yes | 9 | high | Gives fair knowledge on treatment of ankle deformities and in patients with MO. Also, some information on pain with standardized measure, however only on group level. |
| Management of forearm deformities with ulnar shortening more than 15 mm caused by hereditary multiple osteochondromas, 2013, Tang | yes | yes | yes | yes | yes | yes | yes | yes | no | yes | 9 | high | Contributes to knowledge on treatment of forearm deformities and pain in patients with MO. Study- specific, structured assessment methods used for pain and activities of daily living pre and postoperatively. |

| **Title, year, first author**  **Cross-sectional studies** | **1. Were the criteria for inclusion in the sample clearly defined?** | **2. Were the study subjects and the setting described in detail?** | **3. Was the exposure measured in a valid and reliable way?** | **4. Were objective, standard criteria used for measurement of the condition?** | **6. Were confounding factors identified?** | **7. Were strategies to deal with confounding factors stated?** | **7. Were the outcomes measured in a valid and reliable way?** | **8. Was appropriate statistical analysis used?** | **Number yes and not applicable** | **Quality** | **Contribution to knowledge om chronic pain and fatigue.** |
| --- | --- | --- | --- | --- | --- | --- | --- | --- | --- | --- | --- |
| The assessment of fatigue and pain in multiple osteochondromas: A Dutch Cohort Study, 2022, Amajjar | unclear | unclear | n/a | unclear | unclear | unclear | yes | unclear | 2 | Low | This is a conference abstract and therefore gives limited data. The results however are promising and give important addition to the current very limited knowledge on fatigue and pain in MO, in a relatively large cohort. |
| Hereditary Multiple Exostosis and Pain, 2005, Darilek | yes | yes | no | no | yes | unclear | yes | no | 4 | moderate | Important addition to the current very limited knowledge on pain and qol in children and adults with MO. |
| Health-related quality of life in people with hereditary multiple exostoses, 2012, Chhina | no | no | n/a | no | yes | no | yes | yes | 4 | moderate | Important addition to the current very limited knowledge on pain and qol in children and adults with MO. |
| Use of Pediatric Outcomes Data Collection Instrument to Evaluate Functional Outcomes in Multiple Hereditary Exostoses, 2023, De Oliveira | yes | no | n/a | yes | no | no | yes | unclear | 4 | moderate | Important addition to the current very limited knowledge on the functional levels and pain in paediatric patients with MO. |
| Osteochondromatosis: clinical variability and factors related to quality of life in children and adults, 2022, Caino | no | yes | n/a | yes | unclear | no | yes | yes | 5 | moderate | Important addition to the current very limited knowledge on pain and qol in children and adults with MO. |
| Natural History of Multiple Hereditary Osteochondromatosis of the Lower Extremity and Ankle, 2002, Noonan | no | yes | n/a | yes | no | no | yes | yes | 5 | moderate | Gives fair contribution to knowledge on ankle involvement and pain in adults with MO who had not had corrective surgery. Several validated pain measures used. |
| Evaluation of the Forearm in Untreated Adult Subjects with Multiple Hereditary Osteochondromatosis, 2002, Noonan | yes | yes | n/a | yes | no | no | yes | yes | 6 | moderate | Gives fair contribution to knowledge on forearm deformity and pain in adults with MO who had not had corrective surgery. Several validated pain measures used. |
| The impact of hereditary multiple exostoses on quality of life, satisfaction, global health status, and pain, D'Ambrosi, 2017 | yes | yes | n/a | yes | unclear | unclear | yes | yes | 6 | moderate | Important addition to the current very limited knowledge on pain and quality of life in children and adults with MO. |
| Fatigue and pain in children and adults with multiple osteochondromas in Norway, a cross-sectional study, 2018, Bathen | yes | yes | n/a | yes | no | n/a | yes | yes | 7 | high | First study that shows that fatigue is a problem in MO patients, contributes to knowledge on pain in MO. |
| Pain, Physical and Social Functioning, and Quality of Life in Individuals with Multiple Hereditary Exostoses in the Netherlands, 2012, Goud | yes | yes | n/a | unclear | yes | yes | yes | yes | 7 | high | Important addition to the current very limited knowledge on pain and quality of life in children and adults with MO |
| The Impact of Isolated Versus Multiple Osteochondromas: Analysis of the CoULD Registry, 2022, Wessel | yes | yes | n/a | yes | yes | unclear | yes | yes | 7 | high | Gives fair contribution to knowledge on forearm deformity and pain in adults with MO. Several validated pain and function measures used. |
| Functional Impairment of Hip Joint and Activities of Daily Living Failure in Patients with Multiple Hereditary Exostoses, 2022, Matsumoto | yes | yes | n/a | yes | yes | yes | yes | yes | 8 | high | The study gives important addition to the current very limited knowledge on pain, function and activities of daily living in adults with MO. |

| **Title, year, first author**  **Qualitative study** | **1. Is there congruity between the stated philosophical perspective and the research methodology?** | **2. Is there congruity between the research methodology and the research question or objectives?** | **3. Is there congruity between the research methodology and the methods used to collect data?** | **4. Is there congruity between the research methodology and the representation and analysis of data?** | **5. Is there congruity between the research methodology and the interpretation of results?** | **6. Is there a statement locating the researcher culturally or theoretically?** | **7. Is the influence of the researcher on the research, and vice- versa, addressed?** | **8. Are participants, and their voices, adequately represented?** | **9. Is the research ethical according to current criteria or, for recent studies, and is there evidence of ethical approval by an appropriate body?** | **10. Do the conclusions drawn in the research report flow from the analysis, or interpretation, of the data?** | **Number yes and not appliccable** | **Quality** | **Contribution to knowledge om chronic pain and fatigue.** |
| --- | --- | --- | --- | --- | --- | --- | --- | --- | --- | --- | --- | --- | --- |
| Hereditary multiple exostoses: a qualitative study exploring families’ and patients’ perceptions of disease impact and self-expressed needs, 2000, Frazer | no | yes | unclear | yes | yes | no | no | yes | no | yes | 5 | moderate | Contributes to knowledge on patients experience of pain and impact of the condition. |
